# Cell Membrane-Coated Nanoparticles as Biomimetic Drug Delivery Systems: Design, Therapeutic Applications, and Translational Challenges

**DOI:** 10.3390/pharmaceutics18101314

**Published:** 2026-10-16

**Authors:** Shery Jacob, Namitha Raichel Varkey, Rekha Rao, Pottathil Shinu, Razia Khanam

**Affiliations:** 1 Department of Pharmaceutical Sciences, College of Pharmacy, Gulf Medical University, Ajman P.O. Box 4184, United Arab Emirates; 2025mdd01@mygmu.ac.ae; 2 Department of Pharmaceutical Sciences, Guru Jambheshwar University of Science and Technology, Hisar 125001, India; rekhaline@gjust.org; 3 Department of Biomedical Sciences, College of Clinical Pharmacy, King Faisal University, Al-Ahsa 31982, Saudi Arabia; spottathail@kfu.edu.sa; 4 Department of Biomedical Sciences, College of Medicine, Gulf Medical University, Ajman P.O. Box 4184, United Arab Emirates; drrazia@gmu.ac.ae

**Keywords:** cell membrane-coated nanoparticles, membrane surface engineering, cancer therapy, photothermal therapy, photodynamic therapy, targeted drug delivery

## Abstract

Biomimetic cell membrane-coated nanoparticles (CM-NPs) have emerged as a promising class of nanocarriers that integrate the tunable physicochemical properties of engineered nanoparticles with selected biological features of naturally derived cell membranes. By retaining membrane-associated proteins, lipids, glycans, and receptors, CM-NPs have demonstrated the potential to modulate immune recognition, prolong systemic circulation, improve biocompatibility, and facilitate disease-specific targeting; however, these effects depend on the membrane source, nanoparticle composition, coating integrity, fabrication method, and biological context. This review critically examines major membrane-engineering strategies, including lipid insertion, covalent conjugation, metabolic glycoengineering, and genetic modification, together with fabrication methods such as extrusion, sonication, electroporation, in situ polymerization, and microfluidics. Particular emphasis is placed on nanoparticles coated with erythrocyte, platelet, stem cell, immune cell, bacterial, cancer cell, and hybrid membranes. Their therapeutic applications are critically evaluated, with a primary focus on cancer therapy, including photothermal and photodynamic approaches, alongside applications in cardiovascular, neurological, infectious, inflammatory, and regenerative diseases. Recent CM-NP platforms are systematically summarized according to membrane source, therapeutic cargo or nanoparticle core, disease application, and major outcomes, while recent patents are discussed to highlight emerging technological and translational developments. Despite encouraging preclinical findings, clinical translation remains constrained by challenges in membrane isolation and purification, batch reproducibility, manufacturing scalability, preservation of membrane integrity and functionality, quality control, long-term stability, biosafety, and regulatory standardization. Advances in membrane engineering, hybrid membrane systems, scalable manufacturing, standardized characterization, and rigorous safety evaluation will be important for advancing CM-NPs toward clinical translation.

## 1. Introduction

A fundamental challenge in nano-based drug delivery systems is the design of an effective biointerface, which governs how nanoparticles interact with the biological milieu following administration. Beyond serving as carriers for therapeutic cargo and enabling its controlled release at the desired site, nanoparticles must be engineered to minimize undesirable interactions with biological components while simultaneously promoting selective recognition and accumulation at the target site [1]. Regardless of their promising in vitro performance, successful in vivo translation critically depends on the ability of nanoparticles to navigate complex physiological barriers and establish favourable biointeractions [2].

A major challenge in nanoparticle drug delivery is opsonization, followed by rapid clearance by the mononuclear phagocyte system (MPS). Upon entering the bloodstream, nanoparticles rapidly adsorb plasma proteins, forming a dynamic protein corona [3]. Protein adsorption alters the biological characteristics of nanoparticles, enhancing their recognition and uptake by phagocytic cells through complement and Fc receptors, with these interactions largely governed by physicochemical forces [4]. Furthermore, nanoparticle size and surface curvature play important roles in modulating protein–nanoparticle interactions and the resulting surface biomolecular profile. Consequently, opsonized nanoparticles are rapidly sequestered by MPS located particularly the liver and spleen, leading to accelerated systemic clearance, shortened circulation time, and reduced accumulation at target sites [1]. Furthermore, renal filtration represents an important elimination pathway for ultrasmall nanoparticles, particularly those with hydrodynamic diameters below approximately 5.5–6 nm, which can facilitate efficient glomerular filtration and urinary excretion [5]. Only nanoparticles that successfully evade this clearance mechanisms can reach the target tissue, and the proportion of the administered dose that accumulates at the desired site is referred to as the delivery efficiency [6]. In addition to lowering therapeutic efficacy, extensive MPS uptake may increase off-target drug deposition and systemic toxicity. Therefore, minimizing protein adsorption and evading MPS-mediated clearance have become critical design objectives in nanomedicine.

This narrative review was conducted using a structured literature search of PubMed, Scopus, Web of Science, and Google Scholar, primarily covering studies published from 2010 to 2026, with earlier landmark studies included where relevant. Search terms included combinations of “cell membrane-coated nanoparticles”, “biomimetic nanoparticles”, “membrane coating”, “red blood cell membrane”, “platelet membrane”, “stem cell membrane”, “immune cell membrane”, “cancer cell membrane”, “bacterial membrane”, “drug delivery”, and “surface engineering”. English-language original research articles and relevant reviews were selected based on scientific relevance, methodological quality, and relevance to the scope of CM-NPs. Particular emphasis was placed on recent studies involving a clearly defined nanoparticle core coated with isolated cellular membranes. Cell-derived vesicles used directly as carriers without a synthetic nanoparticle core were excluded from the principal analysis. Studies summarized in the tables were selected to illustrate distinct membrane sources, nanoparticle designs, therapeutic applications, and recent advances.

## 2. Surface Functionalization Strategies

Nanoparticles have been extensively surface-engineered to reduce protein adsorption, minimize opsonization, evade rapid clearance by the MPS, and thereby prolong their plasma half-life in systemic circulation. Stealth strategies, including PEGylation, zwitterionic surface modification, and biomimetic cell membrane coating, are among the most widely investigated approaches for creating a biointerface that limits immune recognition while enhancing nanoparticle stability and delivery efficiency. PEGylation forms a hydrated steric barrier around nanoparticles, thereby reducing protein adsorption, macrophage uptake, and non-specific immune recognition, ultimately extending blood circulation time [7]. Despite its widespread use as a stealth coating, PEGylation has several limitations, particularly its potential to induce immunogenicity, including anti-PEG antibody production, hypersensitivity reactions, complement activation, and accelerated blood clearance (ABC) following multiple-dosing [8,9]. Additionally, PEGylation may reduce cellular uptake due to steric hindrance, a phenomenon frequently named as the “PEG dilemma”. Furthermore, PEG is generally considered non-biodegradable, and repeated or prolonged exposure to PEG-containing therapeutics has been associated with cytoplasmic vacuolation in certain tissues. High-molecular-weight PEG may also exhibit reduced clearance, leading to prolonged tissue retention and potential accumulation [10]. To overcome the limitations of PEGylation, several alternative stealth materials have been investigated, including non-ionic polymers such as poly(N-(2-hydroxypropyl) methacrylamide),polypeptide-based materials, including elastin-like polypeptides and engineered amino acid sequences, owing to their excellent biocompatibility and antifouling properties [11]. Alternatively, zwitterionic surface modification generates a highly hydrated surface through electrostatic interactions, providing excellent resistance to biofouling and protein adsorption [12]. Compared with PEG, zwitterionic coatings exhibit superior long-term stability, reduced immunogenicity, and a lower likelihood of inducing ABC upon repeated administration. Representative zwitterionic polymers include poly(carboxybetaine), poly(sulfobetaine), and poly(2-methacryloyloxyethyl phosphorylcholine), all of which have demonstrated excellent biocompatibility and antifouling properties [13].

In biomimetic nanomedicine, a clear distinction should be made between cell membrane-coated nanoparticles (CM-NPs) and cell-derived vesicular carriers. CM-NPs are hybrid systems in which membranes isolated from source cells are used to coat a synthetic nanoparticle core, thereby combining the physicochemical properties of the core with selected biological features of the source membrane. In contrast, extracellular vesicles (EVs), outer membrane vesicles (OMVs), and other cell-derived microparticles are naturally or biologically generated vesicular structures that can function directly as carriers without a synthetic nanoparticle core. Although these vesicles may undergo surface engineering through lipid insertion, covalent conjugation, metabolic glycoengineering, or genetic engineering [14,15,16,17], they are not classified as CM-NPs in this review. Accordingly, the present review primarily focuses on membrane-coated nanoparticles, while cell-derived vesicles are discussed only where relevant to membrane engineering strategies, biological functions, or comparative aspects of biomimetic drug delivery. Various surface-engineering approaches can enhance targeting, stability, and interactions with biological systems. The key principles, advantages, limitations, and representative applications of these approaches are summarized in Table 1.

The surface-engineering strategies discussed below can be applied at different stages of CM-NP fabrication. Direct approaches, such as lipid insertion and covalent conjugation, may be performed on isolated cell membranes or preformed CM-NPs, whereas metabolic glycoengineering and genetic engineering generally require modification of viable donor cells before membrane isolation and nanoparticle coating. Although some of these strategies are also applicable to EVs and other cell-derived vesicles, such vesicles are not classified as CM-NPs in this review and are discussed only as related or comparative examples of membrane engineering.

### 2.1. Lipid Insertion (Lipid Anchoring)

It is a simple and minimally invasive membrane functionalization strategy in which amphiphilic molecules containing hydrophobic lipid anchors, such as cholesterol, dioleoyl phosphatidylethanolamine (DOPE), 1,2-dimyristoyl-sn-glycero-3-phosphoethanolamine (DMPE) or 1,2-distearoyl-sn-glycero-3-phosphoethanolamine-polyethylene glycol (DSPE), rapidly insert into the phospholipid bilayer through hydrophobic associations [18,19]. Lipid insertion enables rapid surface functionalization while preserving native membrane structure and associated proteins, thereby retaining the biological properties of the source cell. Lipid insertion is applicable to donor cells, isolated cell membranes, and preformed cell membrane-coated nanoparticles; it has also been independently employed for the surface engineering of EVs and other cell-derived vesicles. The efficiency and stability of membrane anchoring depend on several factors, including the lipid anchor structure, acyl chain length, PEG molecular weight, membrane fluidity, incubation temperature, and lipid composition [20]. Lipid-mediated cell surface engineering has emerged as a non-genetic alternative to chimeric antigen receptor (CAR) based approaches by anchoring artificial receptors onto the cell membrane using lipid-PEG conjugates [21]. Among the lipid anchors evaluated, DMPE, DSPE, and cholesterol, DMPE exhibited superior membrane insertion, stability, and retention, highlighting its potential for efficient lipid-based cell surface engineering and targeted cancer therapy. Lipid insertion has been extensively applied in the functionalization of erythrocyte [22], platelet [23], leukocyte [24], cancer cell [21], stem cell [25], bacterial cell [26], and extracellular vesicular membranes [27] for targeted drug delivery, immune modulation, molecular imaging, and cancer immunotherapy. An erythrocyte membrane-derived nanovesicle system was functionalized for targeted paclitaxel delivery to gastric cancer using a lipid insertion strategy [28]. Tumor-targeting anti-EGFR-iRGD proteins were anchored onto the erythrocyte membrane via 1,2-Distearoyl-sn-glycero-3-phosphoethanolamine–polyethylene glycol–maleimide (DSPE-PEG-MA), enabling enhanced tumor targeting and penetration without direct chemical conjugation. The resulting nanovesicles (~172 nm) exhibited good stability, sustained biphasic drug release, prolonged tumor retention (>48 h), and significantly inhibited tumor growth by 61% in a gastric cancer mouse model while minimizing systemic toxicity. Membrane anchor stability is governed by lipid structure, with long-chain covalently linked lipids providing extended membrane retention (>80 days), whereas short-chain and ester-linked derivatives are rapidly eliminated [29]. Furthermore, PEGylation of lipid anchors decreases macrophage uptake, thereby enhancing in vivo circulation longevity. Lipid insertion enables facile surface functionalization of CM-NPs; similar principles have also been independently applied to exosomes and other cell-derived vesicles. Longer C18–C20 lipid chains provide greater membrane stability and retention than shorter chains [30]. Despite its simplicity and biocompatibility, lipid insertion has several limitations, including gradual desorption or exchange of lipid anchors, transient membrane retention, particularly in proliferating cells, and potential alterations in membrane fluidity at high lipid densities. Moreover, it’s in vivo stability is influenced by lipid anchor composition, interactions with serum components, and membrane turnover [20,31].

### 2.2. Covalent Conjugation

In the context of CM-NPs, covalent functionalization involves the attachment of bioactive molecules to isolated cell membranes or membrane-coated nanoparticle surfaces. Similar conjugation chemistry is also used for other nanocarriers, including liposomes and EVs, although these systems are not classified as CM-NPs. Unlike physical adsorption or lipid insertion, covalent conjugation provides stronger and more permanent attachment of targeting ligands, thereby improving surface stability, circulation performance, and targeting efficiency [32]. This method generally involves the reaction between functional groups present on the carrier surface and complementary reactive groups on the ligand. Commonly used functional groups include carboxyl (-COOH), amine (-NH_2_), thiol (-SH), hydroxyl (-OH), maleimide, aldehyde, and azide groups [33]. For example, nanoparticle surface carboxyl groups can be activated by 1-Ethyl-3-(3-dimethylaminopropyl) carbodiimide (EDC)/N-Hydroxysuccinimide (NHS)-mediated carbodiimide chemistry, forming reactive ester intermediates that couple with primary amines on proteins or peptides to generate stable amide bonds. Similarly, maleimide-functionalized surfaces react selectively with thiol-containing ligands to form stable thioether linkages [34]. Thus, stable membrane functionalization can be achieved through covalent approaches such as EDC/NHS coupling, maleimide–thiol reactions, or copper-free click chemistry [35], allowing durable and controlled attachment of diverse bioactive molecules [36]. Covalent conjugation has been widely investigated in targeted drug delivery systems. For instance, antibodies such as anti-HER2, anti-EGFR, or anti-PD-L1 can be covalently linked to nanoparticles to enhance cancer cell-specific targeting [37]. Peptides such as RGD may also be conjugated to the surface of carriers to promote binding to integrin receptors overexpressed on tumor cells and angiogenic vasculature. This approach increases drug accumulation at the target site, reduces off-target toxicity, and improves therapeutic efficacy [38]. Covalent modification can also be applied to biological membranes by exploiting reactive groups naturally present on their surfaces, enabling the attachment of functional ligands without extensive membrane reconstruction. Surface covalent conjugation has emerged as a powerful bacterial surface engineering strategy for modulating bacteria–host interactions in therapeutic applications [39]. However, nonspecific covalent conjugation may alter membrane protein conformation, ligand orientation, or biological activity and often requires additional reaction and purification steps. Careful optimization is therefore essential to achieve stable functionalization while preserving membrane integrity and biological properties.

### 2.3. Metabolic Glycoengineering (MGE)

MGE, also known as metabolic oligosaccharide engineering (MOE) is an emerging cell surface engineering strategy in which cells are metabolically labeled with synthetic monosaccharide analogues bearing bioorthogonal chemical reporters [40]. These analogues are incorporated into newly synthesized cell surface glycans during normal biosynthesis, enabling the subsequent conjugation of functional molecules through bioorthogonal click chemistry. For CM-NP fabrication, MGE is primarily a donor-cell engineering strategy: viable cells are metabolically modified before membrane isolation, allowing the engineered surface features to be transferred subsequently to CM-NPs. MGE has also been independently applied to EV engineering; however, engineered EVs are considered cell-derived vesicular carriers rather than CM-NPs. Unlike conventional chemical conjugation, MGE involves culturing living cells with unnatural monosaccharide analogues, such as tetraacetylated N-azidoacetylmannosamine (Ac_4_ManNAz), tetraacetylated N-azidoacetylgalactosamine (Ac_4_GalNAz), or tetraacetylated N-azidoacetylglucosamine (Ac_4_GlcNAz), which are metabolically processed and incorporated into membrane-associated glycans and glycolipids during normal glycan biosynthesis [41]. These modified glycoconjugates present reactive azide (–N_3_) groups on the cell surface, which can subsequently undergo highly selective and catalyst-free bioorthogonal reactions, particularly strain-promoted azide–alkyne cycloaddition (SPAAC),with dibenzocyclooctyne (DBCO) or bicyclononyne (BCN) functionalized antibodies, peptides, aptamers, fluorophores, nanoparticles, or therapeutic agents [42,43]. This approach enables precise and uniform ligand presentation while preserving membrane integrity and the biological activity of membrane proteins. MGE enables stable, site-specific, and controllable surface functionalization of living cells through endogenous glycan biosynthesis without compromising cell viability or membrane integrity and is broadly applicable across mammalian cell types [44,45,46]. Owing to these advantages, MGE has been extensively employed to impart new biological functions to cells [47], fabricate glycoengineered cell membrane-coated nanoparticles [48], enable in vivo cell tracking [49] and metabolic glycan labelling [50,51], enhance targeted cell and drug delivery [52], and facilitate single-cell encapsulation for regenerative medicine and therapeutic applications [53]. MGE has been widely applied to engineer EVs, stem cells, immune cells, and cancer cell membranes for targeted drug delivery, molecular imaging, tissue regeneration, and cancer immunotherapy. MGE combined with bioorthogonal copper-free click chemistry (BCC) provides a simple and biocompatible strategy for engineering EV surfaces. MGE has been combined with cancer cell membrane-camouflaged liposomes to achieve selective glycan labeling of homologous cancer cells [54]. Compared with conventional surface modification methods, MGE provides excellent site specificity, minimal membrane perturbation, homogeneous ligand distribution, and prolonged surface display under physiological conditions, as functional groups are biosynthetically incorporated into newly synthesized cell-surface glycans rather than being randomly attached to membrane proteins or lipids [40,55]. However, its dependence on active cellular metabolism requires modification of viable donor cells before membrane isolation, prolonged incubation for glycan biosynthesis, and additional processing steps, limiting its applicability to isolated membranes and preformed membrane-coated nanoparticles [56].

### 2.4. Genetic Engineering

The method is an indirect surface functionalization strategy in which donor cells are genetically modified before membrane isolation to express specific membrane proteins or targeting ligands [57]. Gene-editing technologies, including CRISPR/Cas9 and viral vector-mediated transfection, enable the expression of therapeutic proteins, tumor-targeting peptides, cytokine receptors, immune checkpoint proteins (e.g., PD-1 and PD-L1), and anti-phagocytic molecules such as CD47 on the cell surface. Consequently, genetically engineered cell membrane-coated nanoparticles (GCMNs) inherit these bioactive surface proteins in their native orientation, enabling precise and reproducible surface functionalization without post-isolation chemical modification. This strategy enhances receptor-specific targeting, immune modulation, cellular uptake, and therapeutic efficacy, making GCMNs promising platforms for cancer immunotherapy and targeted drug delivery. Cancer cell membrane-coated ZIF-8 nanocarriers facilitated homotypic tumor targeting and selective CRISPR/Cas9 delivery, enhancing genome-editing efficiency and tumor accumulation for precision gene therapy. In another study, CXCR4-overexpressing neural stem cell (NSC) membrane-coated poly(lactic-co-glycolic acid) (PLGA) nanoparticles achieved enhanced targeting of ischemic brain tissue via CXCR4–SDF-1 chemotactic interactions [58]. This strategy increased brain accumulation and enhanced the therapeutic efficacy of glyburide in stroke models, highlighting the promise of genetically engineered cell membranes for targeted central nervous system drug delivery. Extending this strategy, PD-1-overexpressing nanovesicles (P-NVs) derived from genetically modified NSCLC cells were engineered to improve tumor-targeted immunotherapy [59]. However, these cell-derived vesicles function as direct carriers and are distinct from CM-NPs. Co-delivery of cyclophosphamide (CP) further enhanced the therapeutic effect by disrupting the PD-1/PD-L1 immune checkpoint, suppressing regulatory T cells (Tregs), and promoting Ki67^+^CD8^+^ T-cell activation, resulting in improved antitumor immune responses. Recent studies have identified ADAR1, an RNA-editing enzyme involved in endogenous RNA sensing, as a key contributor to poor response and resistance to immune checkpoint blockade therapy. To overcome this limitation, a genetically engineered nanovesicle platform (siAdar1-LNP@mPD1) was developed by encapsulating ADAR1 siRNA within lipid nanoparticles and coating them with membranes from PD-1-overexpressing engineered cells [60]. This system simultaneously blocked the PD-1/PD-L1 immune checkpoint axis and delivered siAdar1 to cancer cells, leading to ADAR1 silencing, increased type I/II interferon production, enhanced tumor sensitivity to IFN-γ, and robust antitumor immunity. A modular genetic engineering approach has been developed in which cell membranes are engineered to express SpyCatcher anchors, enabling covalent attachment of SpyTag-functionalized ligands to membrane-coated nanoparticles. This strategy enables controlled protein display while maintaining native membrane orientation and providing consistent surface functionalization [61]. However, high production costs, lengthy manufacturing processes, biosafety concerns, and regulatory complexities remain major barriers to clinical translation [62]. Beyond these conventional surface functionalization strategies, cell membrane coating has emerged as a biomimetic approach for imparting nanoparticles with native cellular functions. These limitations have encouraged the development of alternative surface engineering approaches, particularly biomimetic strategies that exploit the intrinsic biological properties of natural cell membranes.

## 3. Biomimetic Nanoparticles

Biomimetic cell membrane coating has emerged as a strategy for cloaking nanoparticle cores with natural cellular membranes while retaining selected membrane-associated proteins and glycans. Depending on the membrane source, coating integrity, nanoparticle formulation, and biological context, these membrane components may contribute to immune evasion, prolonged circulation, and source-specific interactions with target tissues [2,63]. However, these effects are not inherent to membrane coating and require system-specific experimental validation.

**Figure 1:**
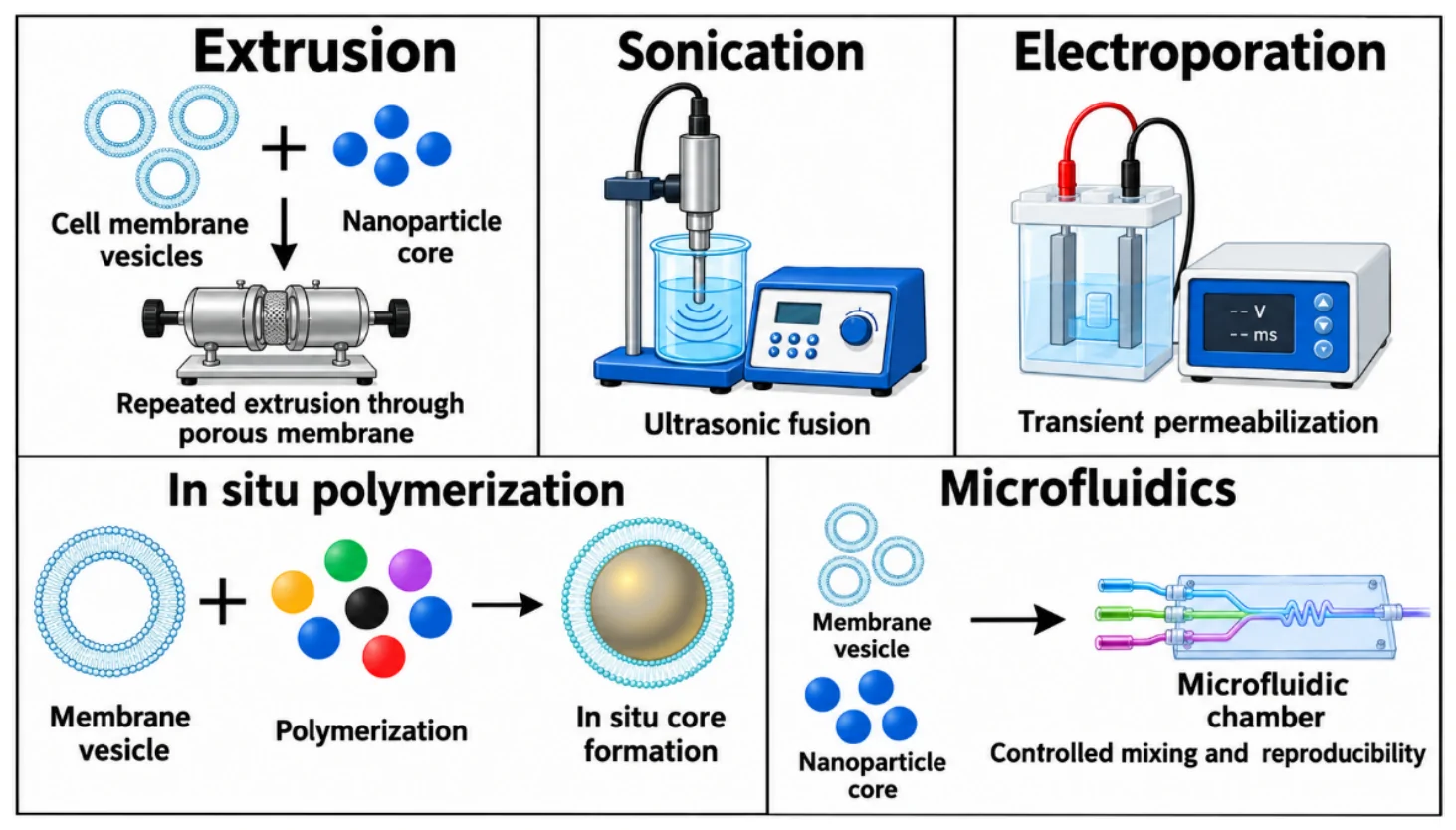
Representative fabrication strategies for biomimetic membrane-coated nanoparticles. Extrusion, sonication, electroporation, in situ polymerization and microfluidic approaches enable the assembly of membrane vesicles with nanoparticle core to generate membrane-coated nanoparticles.

Various fabrication techniques have been explored to assemble isolated cell membrane-derived vesicles around nanoparticle cores to generate CM-NPs, including membrane extrusion, ultrasonic-assisted assembly, electroporation, in situ polymerization, and microfluidic-based coating methods, as illustrated in Figure 1 [2]. Among the available fabrication methods, extrusion and sonication are widely used to integrate nanoparticles with membranes obtained from various cellular sources, such as erythrocytes, platelets, cancer cells, leukocytes, macrophages, stem cells, bacterial and hybrid membranes. Extrusion involves repeatedly passing membrane vesicles and nanoparticle cores through porous membranes, promoting their fusion and generating uniformly coated nanoparticles with consistent particle sizes [64,65]. However, membrane clogging, material loss, and repeated processing can limit scale-up, while mechanical stress may affect membrane integrity and associated proteins. Sonication offers a simpler approach, using ultrasonic energy to facilitate membrane–core fusion with minimal material loss. However, sonication conditions must be carefully optimized, as excessive ultrasonic energy may disrupt membrane integrity, denature membrane-associated proteins, or increase particle-size heterogeneity, potentially affecting the biological functionality and colloidal stability of the coated nanoparticles [66,67,68]. To address some of the limitations associated with conventional coating methods, alternative and emerging fabrication strategies, including in situ polymerization, electroporation, and microfluidic-assisted techniques, have also been explored [69]. In situ polymerization forms nanoparticle cores directly within or in association with membrane vesicles, potentially improving membrane–core interactions and coating efficiency [70]; however, polymerization conditions and residual reagents must be controlled to avoid compromising membrane components. Electroporation uses transient membrane permeabilization to facilitate membrane–core association and can provide controlled assembly, although electrical stress and process optimization may affect membrane integrity and protein functionality. Microfluidic-based fabrication enables precise control over mixing and processing parameters, resulting in more uniform particle sizes, consistent membrane coating, and improved potential for scalable production [71]. However, microfluidic fabrication requires specialized equipment and careful process optimization, and shear forces may affect membrane-protein integrity. Overall, extrusion and sonication are well-established and relatively simple but may be limited by scalability and membrane damage. Microfluidic methods offer better process control, reproducibility, and scale-up potential, whereas electroporation and in situ polymerization provide alternative coating strategies that require careful optimization to preserve membrane proteins and biological functionality [69,71]. Table 2 compares the fabrication techniques in terms of coating quality, membrane functionality, material recovery, and production capacity. Complete coating, correct protein orientation, and GMP suitability require formulation-specific validation.

Membrane association, coating completeness, and preservation of biological function require distinct evidence. Transmission electron microscopy (TEM), increased hydrodynamic diameter, membrane-like zeta potential, and membrane–core colocalization support membrane association but do not confirm complete coverage of every particle. A membrane-impermeant dithionite quenching assay using fluorescently labeled cores and a detergent-disruption control revealed that fewer than 20% of particles were fully coated across the tested conditions [74]. Cryo-TEM, where feasible, can complement conventional TEM to assess shell continuity in hydrated particles, but representative images do not establish complete coating across the population. Quantitative core-accessibility assays should therefore include bare-core, intact-coating, and detergent-disrupted controls, with validated probe exclusion and quenching efficiency [74]. Coating efficiency remains formulation-specific. Fluorescence resonance energy transfer (FRET) can support membrane mixing or fusion in hybrid coatings, as demonstrated for erythrocyte–platelet membranes, but a FRET signal alone does not establish continuous coverage or absence of exposed core [66]. Coating efficiency remains formulation-specific. Membrane orientation and protein retention require separate assessment. In erythrocyte membrane-coated PLGA nanoparticles, domain-specific CD47 immunogold labeling supported extracellular-domain exposure and predominantly right-side-out orientation [75]. Right-side-out orientation and complete membrane coverage were demonstrated for negatively charged polymeric cores under specific conditions [76], but cannot be generalized across membrane sources or fabrication methods. Right-side-out orientation should be assessed using antibodies directed against extracellular and intracellular domains on intact particles, with permeabilized particles and isotype controls to distinguish membrane sidedness from epitope loss. Domain-specific immunogold labeling or antibody-capture assays provide complementary evidence; CD47 antibody-capture assays have also been reported for platelet membrane-coated nanoparticles [23,75]. Dithionite quenching of core-bound fluorophores assesses barrier integrity and should not be interpreted as an orientation assay without a validated leaflet-specific labeling design. SDS-PAGE compares overall protein profiles, while Western blotting or quantitative proteomics assesses selected markers, such as CD47 and, where relevant, P-selectin/CD62P. Marker abundance should be normalized to membrane input or particle number and compared with the source membrane; hybrid coatings should retain markers from each contributing source [23,66,75]. Surface accessibility can be assessed using calibrated small-particle flow cytometry or validated bead-capture assays. Direct particle measurements require documented detection limits, antibody-only controls, and dilution tests to exclude coincident events. Small-particle calibration and reporting principles from MIFlowCyt-EV may guide these measurements, although that framework was developed for EVs rather than CM-NPs [77]. Protein detection or antibody binding alone does not establish biological activity.

Free membrane vesicles and fragments must be distinguished from membrane bound to nanoparticle cores, because residual vesicles can contribute to protein bands, fluorescence signals, and biological responses. Purification should therefore be followed by analysis of both the particle fraction and the separated supernatant or vesicle fractions. Depending on core density and composition, differential or density-gradient centrifugation and size-exclusion chromatography may be suitable, provided that separation is validated. Orthogonal measurements of core recovery, membrane lipids or proteins, and core–membrane colocalization can help assess residual free membrane. Biological-fluid stability requires testing beyond storage in buffer. The stability of erythrocyte membrane-coated PLGA nanoparticles was evaluated in 100% fetal bovine serum over 4 h using turbidity measurements, with bare PLGA and PEG-coated nanoparticles included as controls [78]. This supports resistance to aggregation under the tested conditions, but does not by itself confirm persistence of an intact coating or retained receptor activity. Evaluation in relevant serum or plasma should specify concentration, temperature, and incubation time and monitor particle size, dispersity, membrane detachment, and function after exposure, with appropriate medium controls. Batch-to-batch reproducibility should be assessed using independently manufactured batches, reporting variation in coating completeness, orientation, marker abundance, residual free membrane, colloidal stability, and biological activity. Functional preservation requires assays matched to the intended membrane activity and appropriate comparators. For erythrocyte-coated particles, reduced macrophage uptake provides functional evidence, while CD47 blocking helps assess the contribution of this specific protein [75]. Prolonged circulation in mice provides complementary in vivo evidence [78]. Targeting should be validated using appropriate receptor controls and comparisons with uncoated cores or free membranes. Enhanced uptake alone does not confirm complete coating, orientation, purity, stability, or reproducibility unless directly assessed. Collectively, these parameters should be incorporated into quality-control strategies alongside particle size, PDI, and zeta potential to ensure reproducible coating integrity, membrane functionality, storage stability, and batch-to-batch consistency of CM-NPs.

## 4. RBC Membrane-Coated Nanoparticles

Native red blood cells (RBCs) exhibit excellent biocompatibility, biodegradability, and immune tolerance and remain in systemic circulation for approximately 120 days under physiological conditions [79]. These distinctive biological characteristics have inspired the development of RBC membrane-coated nanoparticles (RBC-NPs), which integrate the biological functionality of natural erythrocyte membranes with the tunable physicochemical properties of synthetic nanocarriers [80]. The membrane-coating strategy can be applied to diverse nanoparticle cores, including polymeric, lipid-based, mesoporous silica, and inorganic nanomaterials, thereby providing considerable flexibility in drug loading, controlled release, and incorporation of diagnostic or therapeutic agents [80,81]. The in vivo performance of RBC-NPs is strongly influenced by their physicochemical properties, particularly particle size, with smaller RBC membrane-coated PLGA nanoparticles exhibiting prolonged circulation and larger particles showing greater hepatic accumulation [82]. Studies have shown that RBC membrane coating can prolong systemic retention relative to uncoated or PEGylated nanoparticles; however, this effect depends on particle size, membrane integrity, coating efficiency, and formulation characteristics. In addition to enhancing circulation longevity, the cell membrane shell offers a biologically compatible surface, whereas the underlying nanoparticle core preserves the therapeutic payload and supports its controlled or sustained release [80]. Consequently, RBC-NPs have been extensively explored for the delivery of chemotherapeutic drugs, proteins, nucleic acids, and imaging agents, as well as for photothermal and photodynamic therapies. Prolonged systemic residence may increase opportunities for accumulation at pathological sites, although the extent of accumulation depends on the formulation and disease model [81].

The lifespan of native RBCs should not be equated with the circulation time of RBC-NPs, because nanoscale geometry, coating defects, and core properties influence clearance. The principal rationale for unmodified RBC membranes is circulation support rather than intrinsic tumor-specific recognition; in the examples discussed below, iRGD, anti-LDLR functionalization, or a MUC1 aptamer provides additional targeting [83,84,85]. Consequently, increased tumor accumulation should be distinguished from receptor-mediated targeting, and the contribution of each modification should be evaluated against the same core with an unmodified RBC coating.

### 4.1. Mechanism of Immune Evasion and Prolonged Circulation

**Figure 2:**
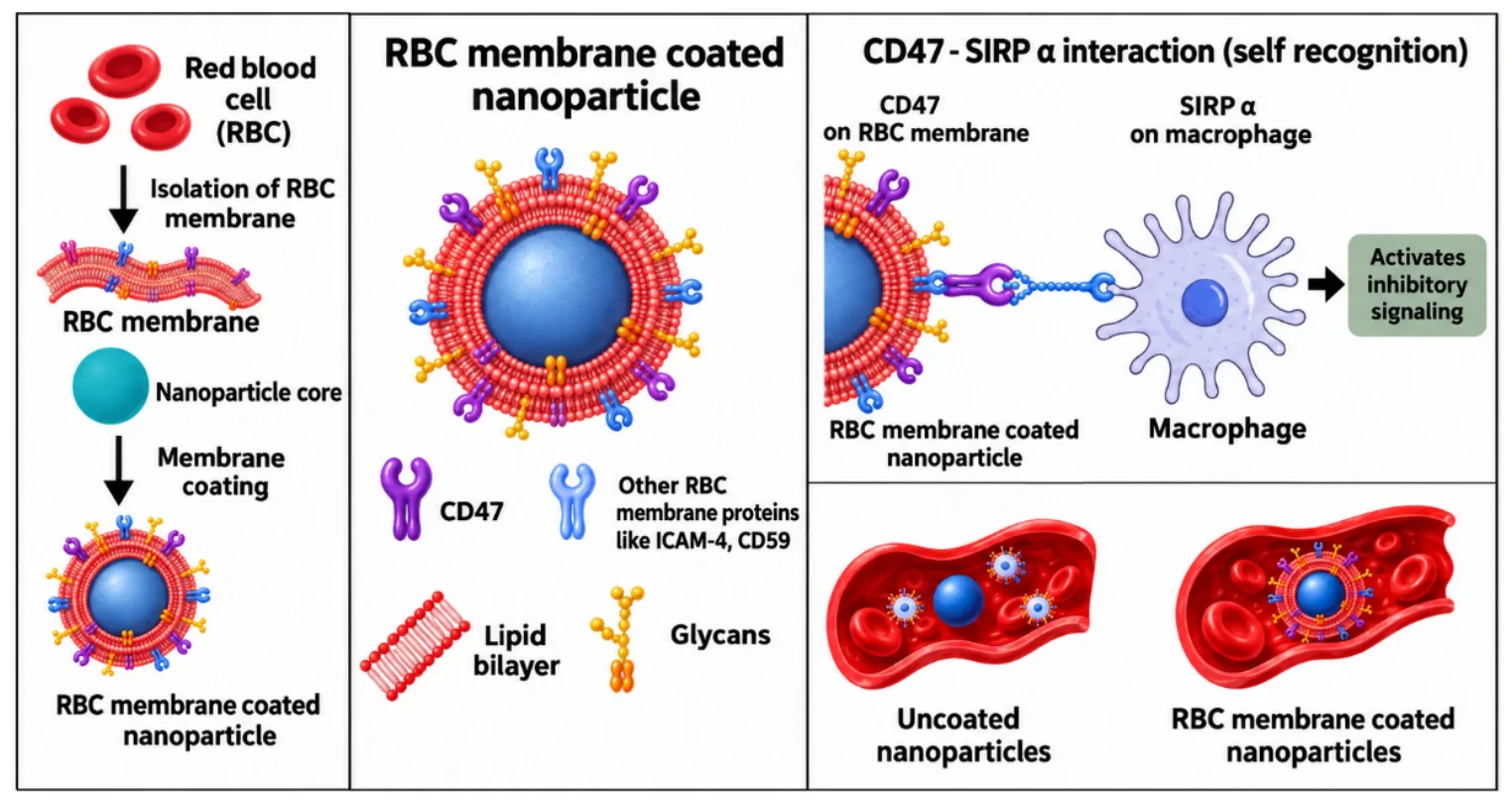
Schematic representation of RBC-NPs, illustrating RBC membrane isolation, coating of the nanoparticle core, and the resulting biomimetic surface that facilitates immune evasion and prolonged systemic circulation.

The prolonged circulation of RBC-NPs is primarily mediated by the transfer of native erythrocyte membrane proteins to the nanoparticle surface. In particular, CD47 acts as a self-recognition signal by binding to signal regulatory protein-α (SIRPα) on macrophages, activating inhibitory signaling that limits phagocytic uptake and subsequent clearance by the MPS [78,79,82]. The RBC membrane coating further provides a biologically compatible interface that reduces nonspecific immune recognition, complement activation, and nanoparticle–immune cell interactions [79,82]. Together, these mechanisms may enable RBC-NPs to reproduce selected biological features of native erythrocytes, potentially reducing immune recognition and extending systemic circulation [78,82]. The key features underlying the immune evasion and prolonged circulation of RBC-NPs are illustrated in Figure 2.

Reduced macrophage uptake does not establish complete immune invisibility or safety after repeated dosing. Complement activation, cytokine responses, hemocompatibility, and clearance should be assessed independently, including with human biological fluids. Source compatibility also matters: comparisons of leukocyte-derived coatings have shown that membrane origin can alter immunological responses, cautioning against generalizing results from matched animal membranes to allogeneic human formulations [86].

### 4.2. Application in Cancer Therapy

iRGD (internalizing RGD) is a tumor-penetrating peptide that initially binds to αv integrins overexpressed on tumor cells and tumor-associated vasculature and subsequently interacts with neuropilin-1, thereby promoting deeper tissue penetration and enhanced intratumoral delivery of therapeutic agents [87]. An iRGD-modified RBC membrane-coated mesoporous silica nanoparticle loaded with doxorubicin (iRGD-RM-(DOX/MSNs)) was developed to enhance tumor-specific drug delivery in triple-negative breast cancer (TNBC) [83]. The RBC membrane conferred immune evasion and prolonged circulation, while the iRGD peptide improved tumor penetration and cellular uptake. The biomimetic nanoplatform demonstrated excellent biocompatibility, reduced macrophage phagocytosis, enhanced uptake by TNBC cells, and superior antitumor efficacy in both in vitro and in vivo models, highlighting its potential as an effective targeted therapeutic strategy for TNBC. Tumor hypoxia is a major barrier to the effectiveness of radiotherapy (RT), as oxygen is essential for radiation-induced tumor cell damage. To overcome this limitation, an artificial nanoscale RBC system was developed by encapsulating oxygen-carrying perfluorocarbon (PFC) within poly(lactic-co-glycolic acid) (PLGA) nanoparticles and coating them with an RBC membrane (PFC@PLGA-RBCM) [88]. RBC membrane encapsulated biomimetic nanoparticles exhibited prolonged circulation, efficient oxygen loading, and enhanced tumor penetration due to their nanoscale size, effectively alleviating tumor hypoxia after intravenous administration. Consequently, oxygen delivery significantly improved the therapeutic efficacy of radiotherapy, highlighting the potential of this RBC-mimetic nanoplatform for hypoxia-targeted cancer treatment. MicroRNAs (miRNAs) are small non-coding RNAs that regulate gene expression and represent promising therapeutic agents for cancer treatment; however, their clinical application is limited by poor stability, rapid degradation, and non-specific biodistribution [89]. To address these limitations, RBC membrane-coated hybrid nanoplatforms have been investigated as carriers for protecting and delivering miRNAs [90]. In one such approach, a biomimetic system (RHAuNCs-miRNA) was developed by coating miRNA-loaded functionalized gold nanocages (AuNCs) with an RBC membrane. The nanoplatform protected miRNAs from enzymatic degradation, provided sustained release and photothermal properties, and enhanced cellular uptake, which was further improved by mild near-infrared (NIR) irradiation. Furthermore, the RBC membrane coating prolonged systemic circulation, promoted tumor accumulation, and improved miRNA delivery, supporting the potential of this approach for cancer gene therapy. Collectively, these studies indicate the potential of RBC- NPs for cancer therapy, although their circulation, tumor accumulation, and therapeutic performance vary with the nanoparticle core, membrane modification, therapeutic cargo, and experimental model.

### 4.3. Photothermal Therapy (PTT)

PTT is a non-invasive therapeutic approach uses photothermal agents to convert near-infrared (NIR) light into heat, enabling precise localized tumor ablation while minimizing systemic toxicity [91]. RBC membrane coating has been employed to enhance the therapeutic efficacy of gold nanoclusters (AuNCs) for PTT [92]. The biomimetic RBC membrane significantly prolonged blood circulation and enhanced tumor accumulation while preserving the NIR-responsive photothermal properties and colloidal stability of AuNCs. RBC-coated AuNCs enhanced photothermal tumor ablation, achieving 100% survival of tumor-bearing mice for at least 45 days after PTT, highlighting the improved therapeutic efficacy of RBC membrane camouflage. RBC membrane-coated natural melanin nanoparticles have been developed as biodegradable photothermal nanoplatforms for cancer treatment [93]. The RBC membrane extended systemic circulation and promoted tumor accumulation while retaining the high NIR photothermal conversion efficiency (~40%) and photoacoustic imaging properties of melanin nanoparticles. Consequently, RBC-coated melanin nanoparticles exhibited significantly improved photothermal tumor ablation in A549 tumor-bearing mice compared with uncoated melanin nanoparticles, highlighting their potential as a biocompatible platform for PTT. RBC membrane-coated polypyrrole nanoparticles (PPy@RBC NPs) exhibited prolonged circulation and enhanced tumor accumulation while retaining their photothermal properties [94]. Co-administration of the endothelin A receptor antagonist BQ123 further increased tumor vascular perfusion, resulting in significantly improved photothermal therapy compared with RBC-coated or PEGylated nanoparticles alone.

Further advances in PTT have incorporated hybrid membrane coatings and combination therapeutic strategies. Hybrid melanin nanoparticles coated with fused RBC and MCF-7 cancer cell membranes combined extended circulation with homotypic tumor-targeting properties [95]. The optimized RBC:MCF-7 membrane ratio (1:1) achieved superior tumor localization and increased photothermal therapy in comparison to pristine melanin nanoparticles and other membrane compositions. Additionally, the nanoparticles retained strong photoacoustic imaging capability, highlighting hybrid membrane coating as an effective strategy to enhance the therapeutic and imaging performance of biomimetic nanomedicines. An RBC membrane-coated, sorafenib-loaded MXene–gold nanocomposite (SF-MX-Au@RBCs) was developed for combined chemotherapy and NIR-triggered photothermal therapy of hepatocellular carcinoma [96]. The nanocomposite (~65 nm) enabled controlled sorafenib release under NIR irradiation and demonstrated enhanced cytotoxicity against HepG2 cells (IC_50_ = 7.3 μg/mL), inducing significant apoptosis and necrosis. Mechanistically, SF-MX-Au@RBCs suppressed MEK/ERK signaling and reduced the expression of angiogenesis-related proteins, including VEGFR2, VEGFR3, and PDGFR. In vivo, the nanoplatform significantly inhibited tumor growth, resulting in a tumor volume of 140 mm^3^ and weight of 0.16 g. Molecular docking revealed favorable interactions between MXene and key oncogenic proteins, further supporting the potential of this RBC membrane-coated platform for synergistic chemo-PTT.

Beyond PTT, RBC membrane-coated nanoplatforms have also been investigated for photodynamic therapy (PDT) and combined photo-based therapeutic strategies. Engineered RBC membrane-coated nanovesicles (ARISP) co-encapsulating salidroside and indocyanine green (ICG) were designed for hypoxia-targeted PDT against TNBC [84]. Salidroside suppressed HIF-1α expression, thereby mitigating tumor hypoxia and improving PDT efficacy, whereas anti-LDLR functionalization of the RBC membrane enhanced circulation longevity, reduced immune clearance, and facilitated preferential uptake by hypoxic tumor cells. The nanoplatform facilitated imaging-guided treatment, effectively inhibited primary tumor growth and metastatic progression, and showed favorable biocompatibility, highlighting its potential for targeted TNBC therapy. Porphyrin-based metal–organic frameworks (MOFs) have emerged as versatile nanoplatforms for PDT; however, their therapeutic translation remains constrained by insufficient tumor specificity and rapid elimination from systemic circulation [97]. To address these challenges, a RBC membrane-coated, Dox-loaded copper MOF was functionalized with a MUC1 aptamer (Apt-RBC-MOF@DOX) for targeted chemo-photodynamic therapy of breast cancer [85]. The RBC membrane prolonged blood circulation and reduced immune clearance, while the MUC1 aptamer enhanced selective tumor targeting. The biomimetic nanoplatform improved tumor cell uptake and localization, promoted hepatic clearance, and achieved effective tumor suppression through combined chemotherapy and 630 nm light-activated PDT. These results demonstrate the promise of Apt-RBC-MOF@DOX as a targeted nanoplatform integrating chemotherapy and photodynamic therapy for breast cancer treatment. Collectively, these studies indicate that RBC membrane coating can improve circulation, tumor accumulation, and therapeutic efficacy in specific PTT and PDT formulations, although the magnitude of these effects is formulation- and model-dependent.

### 4.4. Other Therapeutic Applications

Beyond nanoparticle-based systems, RBC membrane coating has also been extended to living microorganisms, broadening the scope of biomimetic platforms for therapeutic and diagnostic applications. Beyond CM-NPs, RBC membrane coating has also been explored for living microorganisms as a related biomimetic strategy [98]. RBC membrane-coated bacteria have shown prolonged circulation, reduced macrophage uptake and inflammatory responses, and enhanced tumor colonization in experimental models. RBC membrane-coated bacteria exhibited significantly prolonged blood circulation, reduced macrophage uptake and inflammatory responses, and decreased accumulation in healthy organs. Additionally, they markedly enhanced tumor colonization and imaging efficiency while preserving bacterial viability and bioactivity.

RBC membrane-coated nanoparticles have also been investigated for bacterial infections, combining toxin neutralization with targeted antimicrobial delivery. An erythrocyte membrane-coated nanoparticle (Q@RBC-NP) was developed by encapsulating quercetin within an α-GPC-PLA polymeric core and cloaking it with RBC membranes to simultaneously neutralize bacterial pore-forming toxins (PFTs) and deliver antimicrobial therapy [99]. The biomimetic nanoplatform effectively adsorbed toxins secreted by *Staphylococcus aureus* and *Escherichia coli*, while PFT-induced membrane disruption triggered controlled quercetin release at the infection site. Q@RBC-NP showed potent antibacterial activity against both pathogens, protected mammalian cells from toxin-induced damage, and exhibited excellent biocompatibility, supporting its potential for treating drug-resistant bacterial infections. To improve tissue specificity, RBC membranes can be modified with ligands, peptides, antibodies, or aptamers to enable active targeting while maintaining their intrinsic stealth properties.

RBC membrane-based hybrid nanoparticles have also been explored for immune modulation. Hybrid nanoparticles composed of nano-sized autologous or allogeneic RBC membranes encapsulating porous silicon nanoparticles (PSi NPs) were engineered to deliver the model antigen TRP2 [100]. The antigen was either displayed on the RBC membrane surface or encapsulated within the PSi core, with encapsulated TRP2 inducing greater T-cell apoptosis while maintaining the relative proportions of CD4^+^ and CD8^+^ T cells. These results highlight RBC membrane-coated PSi nanoparticles as a promising immunomodulatory strategy for autoimmune disease therapy. Overall, these studies demonstrate the versatility of RBC membrane-based biomimetic platforms beyond cancer therapy, with promising applications in antimicrobial therapy, toxin neutralization, bioimaging, targeted delivery, and immune modulation. Table 3 summarizes representative RBC membrane-coated nanoplatforms, including experimental models, administration regimens, comparators, quantitative outcomes, and evidence limitations. The evidence remains preclinical, and the absence of an uncoated-core comparator in some studies limits attribution of benefits specifically to the membrane coating.

### 4.5. Safety Scalability and Potential Clinical Use

RBC membrane-coated nanoparticles are primarily investigated for prolonged circulation, immune evasion, toxin neutralization, and targeted drug delivery [75,78]. Safety assessment should address donor screening, blood-group compatibility, and removal of residual hemoglobin, intracellular components, nucleic acids, and infectious contaminants [69,107]. Autologous membranes may reduce incompatibility, whereas allogeneic sources require validated donor-selection and compatibility criteria. Hemolysis, complement activation, coagulation effects, repeated-dose immunogenicity, and core- and cargo-related toxicity should be evaluated separately [101,107]. RBCs provide an abundant membrane source, but scalable manufacturing requires standardized isolation, membrane-to-core ratios, coating completeness, protein orientation, removal of free membrane vesicles, sterility, storage stability, and batch-to-batch consistency [69,107]. Processing should preserve key membrane proteins responsible for circulation and immune interactions, with structural characterization linked to functional potency assays. Potential clinical applications include long-circulating drug delivery, toxin sequestration, cancer therapy, and anti-infective treatment [75,78,101]. Compared with platelet, immune-cell, cancer-cell, or bacterial membranes, RBC coatings are particularly suited to circulation-oriented applications with limited immune stimulation. However, current evidence largely supports preclinical feasibility, and clinical translation requires reproducible manufacturing, human-relevant safety evaluation, and demonstration of membrane-specific benefit over appropriate controls.

## 5. Platelet Membrane-Coated Nanoparticles

Platelet membrane-coated nanoparticles (PM-NPs) are biomimetic nanocarriers that combine a synthetic nanoparticle core with a natural platelet membrane. This coating provides platelet-like biological functions and immune camouflage while retaining the core’s physicochemical properties [23]. PM-NPs preserve key platelet membrane proteins, including CD47, P-selectin, GPIbα, GPIIb/IIIa, and integrins. These proteins extend circulation time, reduce macrophage clearance, and promote selective binding to injured blood vessels, inflamed tissues, circulating tumor cells, and certain pathogens [108]. These intrinsic targeting properties have enabled the development of platelet membrane-coated nanocarriers for site-specific drug delivery and improved therapeutic efficacy [109]. In cardiovascular applications, PM-NPs exploit the natural affinity of platelets for atherosclerotic lesions, enabling targeted imaging and early detection of atherosclerosis [110]. These biomimetic nanocarriers selectively target activated endothelial cells, foam cells, and exposed collagen, enabling accumulation in both early and advanced plaques. When loaded with magnetic resonance imaging (MRI) contrast agents, they enable sensitive, noninvasive visualization of atherosclerotic lesions, demonstrating considerable potential for the early detection, monitoring, and management of cardiovascular disease. Beyond cardiovascular applications, PM-NPs have also been investigated for targeted cancer therapy. Vascular disrupting agents (VDAs) are anticancer therapeutics that selectively destroy established tumor blood vessels, causing rapid vascular collapse, reduced blood flow, and tumor necrosis through nutrient deprivation [111]. Despite their potent initial effects, their clinical efficacy is limited because surviving tumor cells at the tumor periphery can promote regrowth, while treatment-induced hypoxia stimulates angiogenesis, leading to tumor recurrence. Platelet membrane-coated mesoporous silica nanoparticles have been designed for the co-delivery of vascular disrupting agents (VDAs) and anti-angiogenic drugs (AADs) to enhance anticancer therapy [112]. By exploiting the natural affinity of platelet membranes for damaged tumor vasculature, these biomimetic nanocarriers selectively accumulate at tumor sites, simultaneously disrupting tumor blood vessels and inhibiting angiogenesis. This synergistic strategy improves antitumor efficacy while reducing tumor recurrence and off-target toxicity. Overall, PM-NPs provide a versatile platform for exploiting platelet-derived biological interactions, although their circulation, targeting, and therapeutic performance depend on membrane integrity, nanoparticle formulation, and disease context.

Compared with unmodified RBC membranes, platelet membranes offer adhesion-mediated recognition of vascular injury and certain disease-associated substrates, in addition to potential circulation benefits. This makes them candidates for vascular and thrombotic delivery, but platelet adhesion does not establish universal tumor specificity. Unlike cancer-cell membranes, which may favor homotypic recognition, platelet targeting depends on compatible ligands exposed by the lesion. Receptor blocking and comparisons with uncoated cores and RBC-coated counterparts are therefore needed to distinguish platelet-mediated adhesion from nonspecific accumulation [23].

### 5.1. Mechanism of Immune Evasion and Prolonged Circulation

Retention of functional CD47 on PM-NPs may reduce macrophage-mediated clearance through self-recognition pathways, potentially contributing to prolonged systemic circulation [113,114]. More importantly, PM-NPs exploit the natural affinity between platelets and tumor cells for active targeting. Platelets interact with cancer cells through multiple receptor–ligand pairs, including CLEC-2/podoplanin, P-selectin/PSGL-1, integrin α6β1/ADAM9, and integrin αIIbβ3/fibrinogen–αvβ3, which facilitate tumor cell adhesion, vascular extravasation, immune evasion, and metastasis [109,115]. For instance, experimental studies have shown that disruption of the CLEC-2–podoplanin axis significantly suppresses tumor metastasis, highlighting this pathway as a promising therapeutic target [116]. Consequently, inhibitors targeting either podoplanin or CLEC-2 have emerged as potential strategies to reduce tumor progression, metastasis, and cancer-associated thrombosis. In a similar manner, platelet integrin α6β1 promotes tumor metastasis through its interaction with ADAM9 expressed on cancer cells [117]. This receptor–ligand interaction enhances platelet adhesion and activation, facilitating tumor cell extravasation and metastatic dissemination. In addition, integrins from the β3 family members αvβ3 and αIIbβ3, play crucial roles in tumor growth, angiogenesis, invasion, and metastatic dissemination [118]. Consequently, dual targeting of these integrins has emerged as a promising therapeutic strategy, with ligand-mimetic β3 integrin antagonists demonstrating enhanced anticancer potential by simultaneously inhibiting multiple integrin-mediated pathways involved in cancer progression.

Interactions established for living platelets do not demonstrate that every receptor remains accessible or active on PM-NPs. Moreover, the roles of native platelets in metastasis and coagulation warrant assessment of whether a particular coating retains unwanted biological activity. Reduced macrophage uptake or complement activation in autologous plasma, as demonstrated for one formulation [23], does not establish safety in allogeneic recipients or after repeated dosing. Complement, cytokine, platelet-activation, coagulation, and hemocompatibility assays should therefore complement targeting studies.

### 5.2. Application in Cancer Therapy

PM-coated PLGA nanoparticles loaded with docetaxel (PM/PLGA/DTX) enhance lung cancer therapy through prolonged circulation, immune evasion, and active tumor targeting [119]. The biomimetic nanoplatform provides sustained drug release and superior tumor accumulation compared with uncoated nanoparticles, with DTX levels peaking at 12 h and remaining more than two-fold higher after 48 h. This enhanced retention significantly improves tumor growth inhibition while reducing the systemic toxicity of docetaxel, highlighting PM/PLGA/DTX as a promising targeted drug delivery system for lung cancer. Platelet membrane-coated chitosan oligosaccharide–PLGA nanoparticles loaded with bufalin (PLTM-CS-pPLGA/Bu) enhance targeted therapy for hepatocellular carcinoma by combining immune evasion with active tumor targeting [120]. The platelet membrane expresses CD47, which reduces macrophage-mediated clearance, and P-selectin, which facilitates binding to CD44 receptors overexpressed on hepatoma cells. The biomimetic nanoparticles enhance cellular uptake, circulation, and tumor accumulation through active targeting and the EPR effect, improving antitumor efficacy while reducing systemic toxicity. Beyond improving tumor targeting, platelet membrane coating has also been combined with functional excipients to address multidrug resistance (MDR). However, P-glycoprotein (P-gp)-mediated drug efflux remains a major contributor to MDR in chemotherapy. PM-coated, D-α-Tocopheryl polyethylene glycol 1000 succinate (TPGS)-modified PLGA nanoparticles loaded with paclitaxel (PTPNs) have been developed to overcome MDR and enhance tumor-targeted drug delivery [121]. The PM provides immune evasion and active tumor targeting, while TPGS inhibits P-glycoprotein (P-gp)-mediated drug efflux. The biomimetic nanoplatform demonstrated sustained drug release under acidic tumor conditions, enhanced cellular uptake, and a 17-fold lower paclitaxel IC_50_ in multidrug-resistant MCF-7/ADR cells compared with free paclitaxel. In vivo, PTPNs enhanced tumor accumulation and therapeutic efficacy while reducing tumor burden and TNF-α levels, supporting their potential against MDR breast cancer. Collectively, these studies indicate that PM-NPs can improve circulation, tumor accumulation, and therapeutic efficacy in specific formulations, although these outcomes remain dependent on the membrane characteristics, nanoparticle core, therapeutic cargo, and experimental model. Therapeutic gains in these multicomponent systems cannot be attributed entirely to the platelet membrane. For example, the lower paclitaxel IC_50_ may reflect TPGS-mediated efflux inhibition as well as altered delivery [121]. Matched comparisons should maintain the same core, cargo, excipients, dose, and release characteristics while varying the coating. Preclinical tumor accumulation and growth inhibition support formulation-specific activity, but do not establish human efficacy or superiority over RBC-, immune-cell-, or cancer-cell-derived coatings.

### 5.3. Photothermal Therapy

PDT utilizes light-activated photosensitizers to generate reactive oxygen species (ROS), resulting in tumor cell destruction, whereas PTT employs photothermal agents to convert light energy into localized heat for tumor ablation [91,122]. PM-NPs enhance PTT through prolonged circulation, reducing immune clearance, and promoting tumor-specific accumulation. PM-coated polypyrrole nanoparticles co-loaded with doxorubicin (PLT-PPy/Dox) have been developed as a biomimetic platform for targeted chemo-PTT of hepatocellular carcinoma [123]. The PM decreased immune clearance and active tumor-targeting abilities, enabling preferential accumulation at tumor sites following intravenous administration. Upon 808 nm NIR irradiation, the polypyrrole core generates localized hyperthermia and promotes controlled Dox release. This synergistic chemo-PTT suppresses primary tumor growth and metastasis, demonstrating the potential of PM-NPs for hepatocellular carcinoma treatment. In another study, platelet–cancer stem cell (CSC) hybrid membrane-coated iron oxide magnetic nanoparticle, (CSC-P) MNs has been developed to enhance targeted photothermal therapy for head and neck squamous cell carcinoma (HNSCC) [124]. The PM promotes immune evasion through “don’t-eat-me” signals, while the CSC membrane enables homotypic tumor targeting through specific adhesion molecules. Compared with single membrane-coated nanoparticles, (CSC-P) MNs provide prolonged circulation, greater tumor deposition, MRI capability, and pronounced photothermal efficacy. These multifunctional nanoparticles effectively suppress HNSCC tumor growth, particularly in immunocompetent models. Their improved therapeutic efficacy highlights the prospect of hybrid membrane-coated nanoplatforms for site-specific cancer treatment. In contrast to PTT, the efficacy of PDT is strongly dependent on oxygen availability. Tumor hypoxia limits PDT efficacy by reducing oxygen-dependent ROS generation, prompting the development of biomimetic nanoplatforms that improve oxygen availability and tumor-specific delivery. Platelet membrane-coated Fe-doped polydiaminopyridine nanoenzymes co-loaded with indocyanine green and metformin (Fe-PDAP-ICG-Met@PM) have been formulated to overcome tumor hypoxia and enhance PDT [125]. The platelet membrane provides reduced immune recognition and active tumor-specific targeting, while the Fe-PDAP nanoenzyme catalyzes oxygen generation from endogenous H_2_O_2_ and depletes glutathione, thereby increasing ROS production. Concurrently, metformin inhibits mitochondrial oxygen consumption, further alleviating hypoxia and enhancing PDT efficacy. The multifunctional nanoplatform also enables magnetic resonance and fluorescence imaging, resulting in significantly improved tumor inhibition and image-guided cancer therapy. Overall, these studies demonstrate the potential of PM-NPs in PTT and PDT, with therapeutic outcomes influenced by membrane composition, nanoparticle design, tumor microenvironment, and the incorporated therapeutic modality.

The hybrid formulation illustrates a possible combination of platelet-associated interactions and cancer-cell recognition [124], but additional membrane sources also increase manufacturing and safety requirements. Membrane ratios, fusion, accessible protein abundance, and batch consistency must be controlled, and comparisons with both single-source coatings are needed to establish the value of hybridization. Photothermal conversion, oxygen generation, and drug action should be attributed to their respective core or cargo components rather than to the membrane alone.

### 5.4. Other Therapeutic Applications

Beyond cancer therapy, PM-NPs have emerged as promising biomimetic carriers for atherosclerosis treatment by leveraging the natural affinity of PMs for atherosclerotic plaques to enable targeted drug delivery [126]. Rapamycin-loaded PNPs exhibited nearly fivefold higher accumulation in atherosclerotic arterial lesions than uncoated nanoparticles, resulting in enhanced plaque targeting. In apolipoprotein E-deficient mice, this targeted approach attenuated atherosclerosis progression and enhanced plaque stability, supporting the potential of PM-NPs for targeted anti-atherosclerotic therapy. PM-NPs have also been explored for the targeted treatment of thrombotic disorders by utilizing the natural thrombus-targeting ability of platelets. PM-coated argatroban-loaded polymeric nanoparticles (PNPArg) have been developed as a biomimetic platform for targeted thrombosis therapy [127]. The PM enables selective accumulation at thrombotic sites, while the hydrogen peroxide (H_2_O_2_)-responsive polymeric core releases argatroban in the oxidative thrombus microenvironment and simultaneously scavenges excess H_2_O_2_. In preclinical studies, PNPArg effectively reduced oxidative stress and inflammation, protected endothelial and macrophage cells from H_2_O_2_-induced injury, and markedly inhibited thrombus formation in a ferric chloride-induced arterial thrombosis mouse model. The nanoplatform also demonstrated excellent biocompatibility, highlighting its potential as a multifunctional therapeutic system for targeted antithrombotic therapy. PM-NPs have also been explored for wound healing applications, where PMs enhance bacterial targeting and promote tissue repair. A PM-coated Fe/Zn-based metal–organic framework nanozyme (PM@MIL-88B-Fe/Zn) has been developed as a biomimetic antimicrobial platform for wound healing [128]. Zinc incorporation enhanced the nanozyme’s peroxidase-like activity and ROS generation, resulting in broad-spectrum antibacterial activity against Gram-positive and Gram-negative bacteria. The PM coating improved bacterial targeting and biocompatibility, while the nanozyme reduced inflammatory cytokine levels, promoted angiogenesis, and accelerated wound closure in vivo. In addition, the platform demonstrated favorable biodegradability, efficient metabolism, and low toxicity.

Overall, PM-NPs have shown potential for targeted treatment of atherosclerosis, thrombosis, and infected wounds, although the extent of site-specific accumulation and therapeutic benefit varies among formulations and disease models. Selected platelet membrane-coated nanoplatforms are compared by experimental model, administration regimen, comparators, reported outcomes, and evidence limitations. Uncoated-core controls, where included, help assess the membrane’s contribution to therapeutic performance, as depicted in Table 4.

### 5.5. Safety Scalability and Potential Clinical Use

For thrombosis and atherosclerosis, platelet adhesion provides a relevant targeting mechanism, whereas immune-cell membranes may better target inflamed endothelium [23,109]. Unlike bacterial membranes used for immune stimulation, platelet coatings are generally investigated for delivery with reduced immune clearance, although they are not universally non-immunogenic. Stem- and cancer-cell sources permit culture expansion, while platelets depend on blood-derived supply. Compared with RBCs, lower platelet abundance and sensitivity to activation can complicate membrane yield and consistency [137]. Donor screening, antigen compatibility, collection and storage conditions, and residual contaminants require control. Autologous sourcing may reduce mismatch but increases logistical demands, whereas allogeneic production requires validated donor selection. Although platelets lack nuclei, residual nucleic acids and other contaminants still require characterization. Potential applications include delivery to injured vessels, thrombotic lesions, tumors, and infected wounds [23,109]. Clinical development requires reproducible membrane isolation and coating, validated potency, sterility, storage stability, and scalable production [137]. Coagulation effects, repeated-dose immune responses, and core- and cargo-related toxicity require separate evaluation. Current evidence supports preclinical feasibility rather than routine clinical use; therefore, platelet-membrane selection should balance demonstrated targeting benefits against safety, scalability, and manufacturing requirements.

## 6. Stem Cell Membrane-Coated Nanoparticles (SCM-NPs)

SCM-NPs are biomimetic nanocarriers that integrate the physicochemical properties of synthetic nanoparticles with the inherent biological functions of stem cell membranes [138]. This approach involves coating synthetic nanocarriers, including PLGA, liposomes, mesoporous silica, gold, and magnetic nanoparticles, with stem cell-derived membranes, particularly from mesenchymal stem cells (MSCs). SCM-NPs are promising biomimetic platforms for targeted drug delivery and regenerative medicine [139,140,141]. Living MSCs possess self-renewal, multilineage differentiation, and paracrine signaling properties relevant to tissue repair, wound healing, and skin regeneration; these functions should be distinguished from the activities of isolated MSC membranes [142,143]. Bone marrow-derived MSC membrane-coated gelatin nanogels (SCMGs) have demonstrated enhanced stability, tumor targeting, and preferential tumor accumulation in experimental models [144].

SCM-NPs can retain selected surface interactions, but they do not reproduce the self-renewal, differentiation, or sustained secretion of living stem cells. Compared with unmodified RBC membranes, MSC membranes provide a rationale for adhesion-mediated lesion association rather than circulation support alone. However, tumor accumulation does not establish active migration or universal tumor specificity; comparisons with uncoated cores and alternative membrane coatings are needed to define the source-specific advantage [144,145].

### 6.1. Mechanism of Immune Evasion and Prolonged Circulation

**Figure 3:**
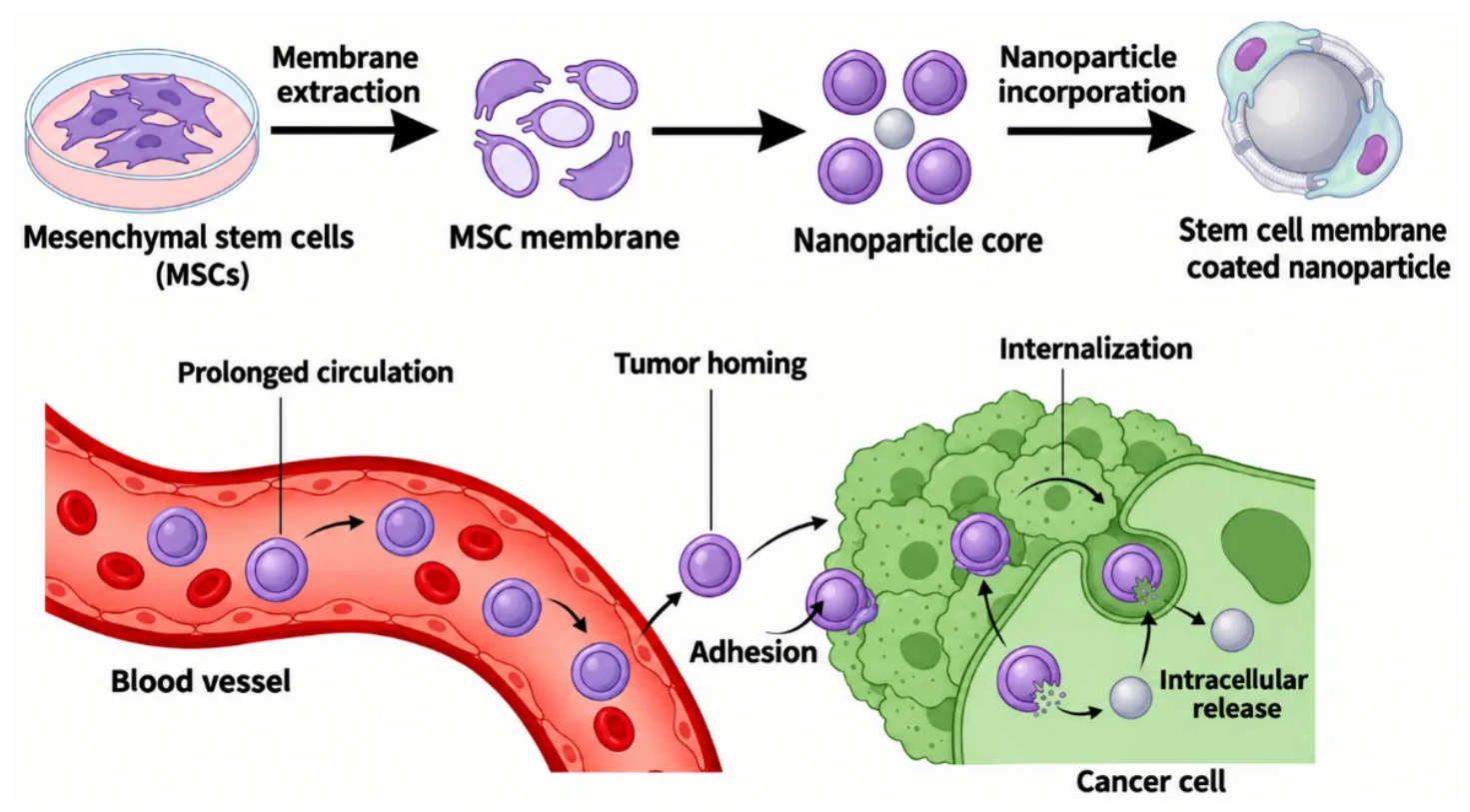
Schematic representation of the fabrication and therapeutic mechanism of SCM-NPs, showing membrane extraction from mesenchymal stem cells, incorporation onto nanoparticle cores, prolonged circulation, tumor homing, cellular adhesion and internalization, followed by intracellular release.

Retention of membrane-associated proteins may enable SCM-NPs to reproduce selected stem cell functions, including immune evasion and disease-site homing, although these effects depend on membrane integrity, nanoparticle characteristics, and the biological model [146]. Stem cell membranes may support lesion-associated interactions and reduced immune clearance, but prolonged circulation, biocompatibility, and targeting must be demonstrated for each SCM-NP formulation. Chemokine receptors retained on the stem cell membrane may further contribute to disease-site targeting through interactions with their corresponding chemokines. For example, CXCR4 recognizes SDF-1/CXCL12, which is highly expressed in several tumors, ischemic tissues, and inflammatory microenvironments [147]. Retained adhesion molecules, including CD44 and VLA-4 (α4β1 integrin), may support binding to activated endothelial surfaces, but particle adhesion and transport across the endothelium require direct confirmation [148,149,150]. Following lesion accumulation, nanoparticles may undergo cellular uptake; the route of entry and subsequent intracellular trafficking depend on the formulation. Stimulus-responsive cargo release requires appropriate core or carrier design and should not be attributed to the stem cell membrane alone. Passive tumor accumulation through the EPR effect is also model-dependent and does not independently establish membrane-mediated targeting. Collectively, these mechanisms may contribute to improved drug accumulation, reduced immune clearance, and therapeutic efficacy, with outcomes varying according to membrane source, formulation, and disease model [139,151,152]. Figure 3 illustrates the integration of stem cell membranes with nanoparticle cores and their subsequent interactions with tumor tissues.

CXCR4–CXCL12 interactions on a coated particle should not be equated with chemotaxis, which requires the signaling and motility machinery of a living cell. Receptor blocking, ligand competition, and receptor-deficient controls can test whether accessible membrane proteins contribute to binding. Likewise, reduced macrophage uptake does not establish complete immune evasion: donor compatibility, complement activation, cytokine responses, and repeated-dose clearance require separate assessment. Low immunogenicity reported for a source MSC population cannot be assumed to persist after membrane isolation, coating, and exposure to human plasma.

### 6.2. Application in Cancer Therapy

MSC membrane-coated nanocarriers have been investigated for tumor delivery, with targeting and toxicity benefits dependent on the formulation and experimental model. Human umbilical cord-derived MSCs (UC-MSC) membrane-coated nanoparticles have emerged as promising biomimetic drug delivery systems for targeted cancer therapy. *UC*-MSC membrane coating has supported tumor accumulation and drug delivery in a preclinical PLGA formulation, providing evidence of formulation-specific targeting rather than universal immune compatibility [145]. Compared with uncoated nanoparticles, MSC membrane-coated PLGA nanoparticles exhibit enhanced cellular uptake, improved tumor accumulation, and increased intracellular delivery of DOX, leading to superior tumor growth inhibition and apoptosis. Small interfering RNA (siRNA) offers a promising gene-silencing approach for cancer therapy, but its clinical application is limited by poor stability, rapid degradation, and inefficient intracellular delivery. To address these limitations, stem cell membrane-coated nanoparticles have been developed as biomimetic carriers for targeted siRNA delivery and combination therapies, including gene silencing and PTT, to enhance anticancer efficacy. MSC membrane-coated Fe_3_O_4_@PDA nanoparticles have been engineered as a biomimetic nanoplatform for the co-delivery of siRNA and PTT. The MSC membrane enhances tumor-targeted siRNA delivery while preserving the magnetic resonance imaging and photothermal properties of the Fe_3_O_4_@PDA core. Delivery of Plk1-specific siRNA effectively silenced gene expression, induced tumor cell apoptosis, and, when combined with PTT, produced superior antitumor efficacy in prostate cancer xenograft models. These results demonstrate the promise of MSC membrane-coated nanoparticles as versatile theranostic carrier combining targeted gene delivery, imaging, and synergistic cancer therapy.

Unlike cancer-cell membranes, which can support recognition of matching tumor cells, MSC membranes are investigated for interactions with lesion-associated signals and adhesion partners. This may broaden the targeting rationale, but does not establish greater selectivity across heterogeneous tumors. Matched core, cargo, dose, and release controls are necessary to separate membrane effects from siRNA action, drug potency, or photothermal activity. The animal findings discussed here support preclinical feasibility and should not be interpreted as evidence of human therapeutic superiority.

### 6.3. Photothermal Therapy

SCM-NPs have gained attention as biomimetic photothermal platforms that integrate stem cell-mediated targeting with efficient light-to-heat conversion for localized therapy. SCM-coated Au–Ag–PDA nanoparticles have emerged as promising photothermal platforms, offering efficient near-infrared light-to-heat conversion and enhanced biocompatibility [153]. Upon 808 nm laser irradiation, Au–Ag–PDA@STCM nanoparticles significantly inhibited sebaceous gland cell proliferation in vitro and reduced sebaceous gland size and sebum secretion in vivo, demonstrating enhanced photothermal therapeutic efficacy with reduced cytotoxicity. These results demonstrate the promise of stem cell membrane-coated photothermal nanoparticles as a targeted therapeutic approach for acne. Similarly, umbilical cord MSC-coated polydopamine nanoparticles loaded with 7-ethyl-10-hydroxycamptothecin (PDA-SN38@SCM NPs) were developed as a targeted platform for the treatment of malignant bone tumors [154]. The stem cell membrane coating reduced macrophage uptake, prolonged blood circulation, and enhanced tumor accumulation, while the polydopamine core retained excellent photothermal conversion efficiency. Drug release was triggered by near-infrared irradiation and the acidic tumor microenvironment, enabling controlled SN38 delivery. In vitro and in vivo studies demonstrated enhanced cellular uptake and synergistic chemo-photothermal effects, highlighting stem cell membrane-camouflaged polydopamine nanoparticles as a promising platform for targeted bone cancer therapy.

Stem cell surface engineering offers an alternative to intracellular nanoparticle loading, enabling higher nanoparticle payloads, reduced cytotoxicity, and improved targeted photothermal therapy. As a related cell-based strategy distinct from SCM-NPs, MSCs have been engineered with surface-attached AuNP/GO hybrid sheets for targeted photothermal deliver. In this context, MSCs were engineered with surface-attached gold nanoparticle/graphene oxide (AuNP/GO) hybrid sheets to overcome the cytotoxicity and nanoparticle leakage associated with intracellular loading [155]. Collectively, these studies highlight the potential of SCM-NPs for photothermal therapy, although their therapeutic performance depends on the stem cell membrane source, nanoparticle composition, and experimental model. The surface-loaded MSC approach uses living cells and therefore has a different mechanism and safety profile from isolated membrane-coated nanoparticles. CAR-T membranes are immune-cell-derived and should likewise be treated as a comparator rather than as stem cell membranes. For PTT, lesion association may be influenced by the coating, whereas light-to-heat conversion originates from the photothermal material; efficacy alone cannot establish retained stem cell membrane function.

### 6.4. Other Therapeutic Applications

MSC membrane-coated mesoporous silica nanoparticles (MSNs) have demonstrated significant therapeutic potential for myocardial infarction by delivering microRNA-21 [156]. This biomimetic nanoplatform enhanced cardiac accumulation, reduced infarct size, and improved cardiac function in experimental myocardial infarction models, supporting its potential for regenerative cardiovascular therapy. MSC membrane-coated nanoparticles have also been explored for the targeted treatment of liver fibrosis by enhancing the delivery of antifibrotic therapeutics to injured hepatic tissue. A MSC-membrane-coated PLGA (MSCM-PLGA) nanoparticle was developed to deliver mitogen-activated protein kinase phosphatase 5 (MKP5), a key regulator of fibrosis [157]. MKP5 deficiency was associated with aggravated hepatic fibrosis through activation of the IRE1/XBP1-mediated endoplasmic reticulum stress pathway, leading to hepatic stellate cell activation and hepatocyte apoptosis. Biomimetic MSCM-PLGA-MKP5 nanoparticles effectively targeted fibrotic liver tissue, suppressed hepatic inflammation and fibrosis, and significantly improved therapeutic outcomes by modulating the IRE1/XBP1 signaling pathway, demonstrating their capacity as a site-specific nanotherapeutic strategy for hepatic fibrosis. MSCM-coated nanoparticles have emerged as promising platforms for neurodegenerative disease therapy by enhancing brain-targeted delivery and neuroprotective effects. MSC-derived neuron-like cell membrane-coated curcumin-loaded PLGA nanoparticles (MM-Cur-NPs) were developed as a biomimetic nanoplatform for Parkinson’s disease (PD) therapy [158]. The neuron-like membrane coating enhances brain targeting and prolongs nanoparticle retention, resulting in increased dopamine levels, improved motor function, and restoration of dopaminergic neurons in PD models. Mechanistically, MM-Cur-NPs reduce oxidative stress, preserve mitochondrial function, inhibit neuronal apoptosis and microglial activation, and suppress neuroinflammation while promoting anti-inflammatory signaling. These results highlight the potential of stem cell membrane-coated nanoparticles as a promising neuroprotective strategy for Parkinson’s disease treatment.

Collectively, these studies illustrate the therapeutic potential of SCM-NPs across diverse disease applications. However, their circulation, tissue targeting, and therapeutic performance depend on the stem cell membrane source, nanoparticle formulation, and experimental model.

### 6.5. Safety Scalability and Potential Clinical Use

MSC membranes offer a potential approach for tumor- or injury-associated delivery, whereas platelet membranes may suit vascular adhesion and immune-cell membranes inflammatory endothelial targeting. RBC membranes primarily support circulation or toxin capture, while bacterial membranes favor antigen delivery and immune stimulation. These distinctions guide source selection rather than establish clinical superiority [145,151]. Compared with RBC or platelet coatings, MSC membranes require culture expansion and purification of nucleated cells, increasing processing complexity and control of residual DNA and contaminants. Donor source, tissue origin, passage number, differentiation state, and culture conditions may alter membrane composition. Production should therefore control cell identity, membrane yield, marker expression, coating integrity, potency, and batch consistency [151,159]. Hybrid membranes additionally require control of source compatibility, membrane ratios, fusion, and receptor accessibility. Safety assessment should exclude viable cells, residual nucleic acids, cellular debris, and potentially immunogenic or growth-promoting components. Removal of viable cells does not establish the safety of the membrane, core, or cargo. Potential applications in cancer, ischemic injury, fibrosis, and neurological disease require indication-specific evidence [145,151]. Clinical translation will depend on scalable manufacturing, sterility, stability, reproducible potency, and repeated-dose safety, with therapeutic effects attributed to demonstrated membrane or cargo mechanisms rather than presumed functions of living MSCs [151,159].

## 7. Bacterial Cell Membrane-Coated Nanoparticles (BCM-NPs)

BCM-NPs are biomimetic nanocarriers formed by coating synthetic nanoparticles with membranes derived from Gram-positive or Gram-negative bacteria [160]. This strategy combines the tunable properties and drug-loading capacity of synthetic nanoparticles with the intrinsic biological functions of bacterial membranes. Retention and accessibility of membrane-associated antigens and immunostimulatory components depend on the bacterial source, isolation, detoxification, and coating process [161]. Consequently, BCM-NPs have been investigated for vaccine development, antimicrobial therapy, and cancer immunotherapy by combining nanoparticle-based delivery with the immunological properties of bacterial membranes.

Membrane-coated synthetic cores should be distinguished from native or engineered OMVs used directly as cargo carriers. OMVs originate from Gram-negative bacteria, whereas Gram-positive bacteria release membrane vesicles without a Gram-negative outer membrane. Evidence from an OMV carrier therefore does not by itself establish the benefit of coating a synthetic core.

### 7.1. Mechanism of Immune Evasion Evasion and Extended Circulation

BCM-NPs and related outer membrane vesicle (OMV)-based platforms integrate the physicochemical advantages of synthetic nanocarriers with the biological and immunostimulatory functions of bacterial membranes. Depending on the bacterial source, these membrane-derived systems contain pathogen-associated molecular patterns (PAMPs), including lipopolysaccharides, lipoproteins, peptidoglycan-associated components, outer membrane proteins, and bacterial nucleic acids, which can be recognized by pattern-recognition receptors such as Toll-like receptors and NOD-like receptors. This recognition activates downstream signaling pathways, including NF-κB, MAPK, and inflammasome-associated signalling, promoting the secretion of inflammatory cytokines, dendritic-cell maturation, macrophage activation, and antigen presentation [162,163]. In preclinical cancer models, selected bacterial membrane platforms have promoted M1-like macrophage polarization, cytotoxic T-cell and natural killer-cell activity, and IFN-γ/CXCL10-associated antitumor responses [164]. Bacterial membrane coatings may retain adhesins and other surface proteins that influence cell binding, but bacterial origin alone does not establish disease-specific targeting. For example, OMV-functionalized Fe_3_O_4_–MnO_2_ nanoparticles were reported to generate oxygen, relieve tumor hypoxia, induce immunogenic cell death, and enhance photothermal immunotherapy [165]. OMV-encapsulated oncolytic adenoviruses induced oxidative stress and excessive autophagy, thereby enhancing viral replication, tumor cell destruction, and antitumor immune responses [166]. These examples combine membrane-mediated immune stimulation with core-, cargo-, or enzyme-dependent effects; oxygen generation, photothermal conversion, and enhanced viral replication should not be attributed to bacterial membrane coating alone. Compared with RBC membranes used for prolonged circulation, bacterial membranes primarily provide antigens and immune stimulation, potentially increasing immune clearance. Platelet and immune-cell membranes offer injury- or inflammation-associated adhesion, whereas bacterial targeting requires validation. In cancer-cell–OMV hybrids, cancer membranes may provide homotypic targeting while bacterial components stimulate immunity; appropriate single-source controls are needed to distinguish these effects.

### 7.2. Application in Cancer Therapy

In cancer therapy, BCM-NPs have been investigated for the delivery of chemotherapeutic agents, photothermal agents, and photosensitizers while exploiting the immunostimulatory properties of bacterial membranes. Whether immune activation improves tumor control without unacceptable systemic inflammation depends on the formulation, dose, route, and model. In vaccine applications, BCM-NPs can function as both antigen-delivery systems and immunostimulatory platforms, with bacterial membrane-associated components providing intrinsic adjuvant activity that can enhance antigen-specific immune responses. Bacterial OMV-based hybrid membrane-coated nanoparticles have also been developed as multifunctional biomimetic platforms integrating tumor-targeting, immunomodulatory, and therapeutic functions. In a recent study, hybrid membrane-coated nanoparticles were developed by fusing breast cancer cell membranes with bacterial OMVs and coating them onto IR780-loaded PLGA nanoparticles [167]. The hybrid formulation promoted tumor accumulation, M1 macrophage polarization, dendritic-cell maturation, and antitumor activity in the reported preclinical experiments. These results support further evaluation for breast cancer bone metastasis but do not establish clinical efficacy or superiority over other membrane sources. The contribution of the hybrid coating must be separated from IR780-mediated sonodynamic effects using uncoated and single-membrane comparators. Another formulation combined a bacterial membrane coating with a PC7A/CpG immunostimulatory core and surface imide groups to enhance radiotherapy [168]. After radiotherapy (RT) induced tumor cell death, BCM-NPs captured released neoantigens and enhanced their dendritic cell uptake and cross-presentation, eliciting robust tumor-specific T-cell responses. Combined BNP–RT therapy promoted tumor regression and durable antitumor immune memory in melanoma and neuroblastoma models. These outcomes reflect the combined effects of radiotherapy, antigen-capture chemistry, the PC7A/CpG core, and bacterial membrane components; their individual contributions require appropriate controls.

### 7.3. Photothermal Therapy

Hybrid membrane-coated nanoparticles incorporating bacterial OMVs and cancer cell membranes have been investigated as multifunctional platforms combining tumor targeting, immune stimulation, and photothermal therapy. In one study, an OMV–cancer cell hybrid membrane was coated onto hollow polydopamine (HPDA) nanoparticles to integrate OMV-mediated immunotherapy with PTT [169]. The hybrid formulation was reported to combine cancer-cell membrane-associated homotypic recognition with OMV-mediated dendritic-cell maturation and HPDA-mediated photothermal ablation. Complete melanoma eradication and limited toxicity in the reported animal experiments are model-specific findings and do not establish general safety or clinical benefit. Comparisons with uncoated HPDA, OMV-only and cancer-cell-only coatings, with and without irradiation, are needed to attribute targeting and efficacy to each component.

### 7.4. Other Therapeutic Applications

BCM-NPs represent promising biomimetic platforms for antibacterial vaccination by combining the immunogenic properties of bacterial membranes with the physicochemical stability of synthetic nanoparticles [161]. In a representative study, OMVs derived from *Escherichia coli* were coated onto 30 nm gold nanoparticles (AuNPs), producing bacterial membrane-coated AuNPs (BM-AuNPs) with enhanced stability under physiological conditions. Upon subcutaneous administration, BM-AuNPs promoted rapid dendritic cell activation and maturation, elicited durable, high-avidity antibody responses, and stimulated robust Th1- and Th17-mediated immunity through increased interferon-γ (IFN-γ) and interleukin-17 (IL-17) production, without enhancing IL-4 secretion. These findings support the potential of BCM-NPs as antibacterial vaccine platforms capable of inducing robust and sustained humoral and cellular immune responses.

Engineered bacterial membrane systems have also been explored for vascular applications, but their effects on atherosclerosis must be assessed separately from their vaccine-related immunostimulatory activity [161,170]. Native pathogen-associated molecular pattern (PAMP)-mediated inflammation cannot be assumed to stabilize plaques or reduce vascular inflammation; any proposed benefit requires source-specific targeting, inflammatory readouts, and vascular safety data. Anti-adhesion therapy instead aims to prevent bacterial attachment without directly killing bacteria, although reduced resistance selection remains a hypothesis requiring evaluation. One approach used polymeric nanoparticles coated with bacterial outer membranes [171]. The resulting outer membrane-coated nanoparticles (OM-NPs) mimic bacteria and competitively inhibit bacterial adhesion to host cells without requiring identification of specific adhesins. Helicobacter pylori-derived OM-NPs demonstrated effective binding to gastric epithelial (AGS) cells. OM-NPs reduced H. pylori adhesion to AGS cells in a concentration- and administration sequence-dependent manner. These findings support a preclinical anti-adhesion mechanism but do not establish infection clearance or clinical efficacy. Recent bacterial membrane-based platforms are summarized in Table 5; the table includes both membrane-coated cores and OMV cargo carriers, whose outcomes should be interpreted according to their distinct architectures and experimental controls.

### 7.5. Safety Scalability and Potential Clinical Use

Bacterial membranes present a distinct safety tradeoff: microbial recognition supports vaccination and cancer immunotherapy but may cause inflammatory toxicity. Lipopolysaccharide (LPS) is a major concern for Gram-negative sources, while Gram-positive bacteria contain other inflammatory components. Detoxification or genetic attenuation should therefore be assessed alongside antigen retention and potency [173,174]. Engineered LPS-free OMVs have shown improved tolerability and anticancer activity compared with wild-type OMVs [174], supporting source engineering while requiring formulation-specific safety evaluation [175]. Bacterial cultivation may offer manufacturing advantages over donor-dependent RBC and platelet sources or expanded stem and cancer cells. However, culture conditions, strain stability, vesicle yield, and purification can affect membrane composition and activity. Scalable production should control strain and culture conditions, remove viable bacteria and contaminants, and distinguish free OMVs from core-bound membranes. Batch testing should assess membrane identity, inflammatory potency, antigen retention, coating completeness, sterility, and stability [173,174]. Hybrid coatings additionally require control of membrane ratios and component-specific activity. Bacterial membranes are primarily suited to antibacterial vaccination, antigen delivery, and cancer immunotherapy requiring controlled immune activation. In contrast, RBC coatings favor circulation-oriented delivery, while platelet, immune-cell, and cancer-cell membranes provide distinct targeting mechanisms. Clinical translation requires evaluation of immunogenicity, biodistribution, clearance, inflammation, and human-relevant safety [173,174].

## 8. Cancer Cell Membrane-Coated Nanoparticles (CCM-NPs)

**Figure 4:**
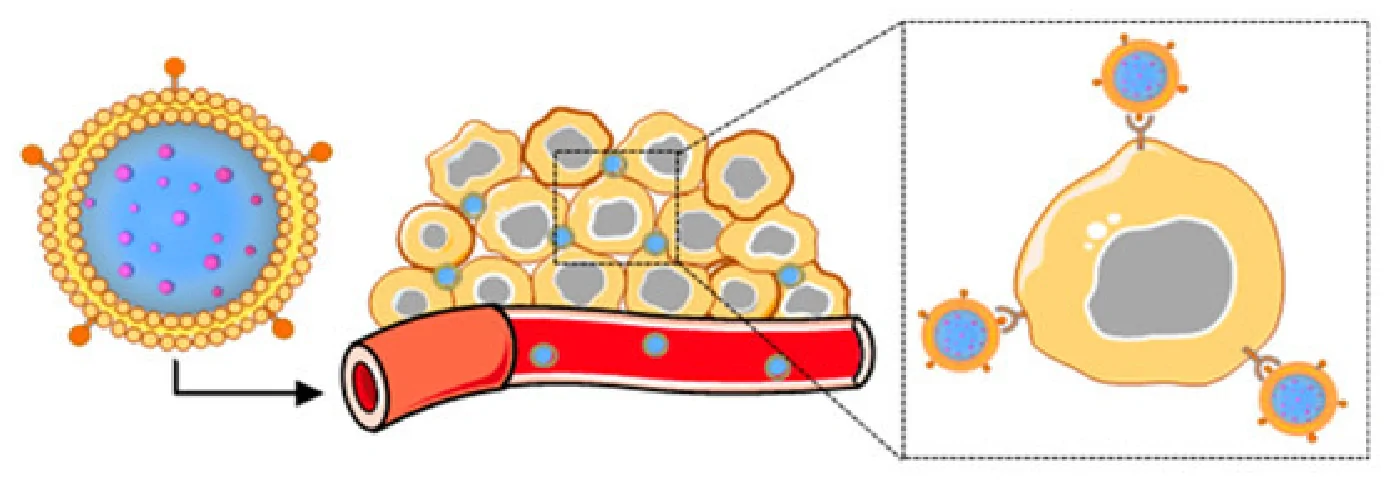
Schematic illustration of the systemic delivery and tumor-targeting mechanism of CCM-NPs. The membrane coating provides self-recognition properties that reduce immune mediated clearance and facilitates homotypic recognition and interaction with tumor cells. Reproduced from Ref. [177].

CCM-NPs are biomimetic nanocarriers formed by coating synthetic nanoparticle cores with cancer cell-derived membranes [176]. By retaining selected membrane proteins and recognition molecules, CCM-NPs may reduce immune recognition and facilitate preferential tumor interactions through homotypic recognition [177] as illustrated in Figure 4. Depending on the membrane source, nanoparticle formulation, and tumor model, CCM-NPs have shown enhanced tumor accumulation and reduced off-target distribution compared with uncoated nanoparticles, supporting their investigation for targeted drug delivery, phototherapy, tumor imaging, and cancer immunotherapy.

### 8.1. Mechanism of Immune Evasion and Prolonged Circulation

Retention of membrane-associated proteins derived from parental cancer cells enables CCM-NPs to recapitulate key features of the native cancer cell surface, thereby potentially reducing recognition and clearance by the MPS [178]. Retained, outward-facing CD47 may bind macrophage SIRPα and reduce phagocytosis, but protein detection alone does not establish functional signaling or prolonged circulation in a coated formulation [179]. Cancer cells may express other immune-modulatory proteins, including PD-L1 and CD24, whose abundance varies among cancer types and subtypes. Their roles in the source cells do not demonstrate equivalent activity on CCM-NPs; retention, orientation, receptor accessibility, and immune effects require formulation-specific assessment [176,180]. In addition, the natural lipid–protein architecture of the cancer cell membrane provides a biomimetic interface that shields the synthetic nanoparticle core from direct interaction with biological components and may reduce nonspecific protein adsorption and subsequent opsonization [179]. Membrane lipids, including phospholipids, cholesterol, sphingolipids, and glycolipids, contribute to membrane organization, fluidity, stability, and nanoparticle–biological interactions [181]. Reduced macrophage uptake in vitro should be distinguished from prolonged blood residence in vivo, and both should be evaluated separately from homotypic binding. Appropriate comparisons include an uncoated core and a non-cancer membrane coating with matched size, dose, and cargo; receptor-blocking or depletion experiments can test whether a retained protein contributes to the observed effect. Compared with RBC membranes used for circulation-oriented delivery, cancer-cell membranes offer homotypic adhesion and tumor antigens. However, targeting may be limited by tumor heterogeneity, source–target mismatch, and culture-induced phenotype changes. Matched and mismatched tumor and normal-cell controls are therefore needed to establish tumor selectivity.

### 8.2. Application in Cancer Therapy

CCM-NPs have been investigated for cancer therapy based on their potential for homotypic tumor targeting, reduced immune clearance, tumor microenvironment modulation, and immunotherapeutic activity. Retention of membrane-associated adhesion and recognition molecules can facilitate homotypic interactions between CCM-NPs and cancer cells, thereby enhancing tumor-specific drug delivery. For example, 4T1 cancer cell membrane-coated PLGA nanoparticles improved tumour accumulation of ABT-737 [182]. Retention of CD47 can suppress macrophage-mediated phagocytosis through CD47–SIRPα signalling, potentially prolonging circulation and enhancing tumour accumulation [179]. CCM coating may also facilitate nanoparticle penetration and drug accumulation within the complex TME, as demonstrated in three-dimensional tumour spheroids [176,183,184,185]. Furthermore, the presence of tumour-associated antigens and neoantigens enables CCM-NPs to function as biomimetic nanovaccines, particularly when combined with immunostimulatory cores or adjuvants [186,187]. These properties also support their application in personalized cancer immunotherapy, with tumour membrane-derived nanovaccines demonstrating enhanced antigen presentation, antitumour immunity, and suppression of tumour recurrence and metastasis [188]. The applications of CCM-NPs have been widely studied across various cancer types, including glioblastoma, breast, gastric, cervical, and oral cancers. In glioblastoma, cancer cell membrane camouflage can facilitate tumor-specific recognition and accumulation through homotypic interactions; however, effective delivery across the blood–brain barrier (BBB) remains an important challenge and may require additional targeting or delivery strategies [189]. Similarly, in breast cancer, CCM-NPs have been investigated to enhance tumor-targeted drug delivery, improve therapeutic accumulation, and reduce off-target exposure. Paclitaxel (PTX)-loaded poly (lactic acid) nanoparticles coated with 4T1 cancer cell membranes exhibited homotypic tumour targeting, enhanced tumour accumulation and biocompatibility, and significant tumour growth inhibition in mice [190].

**Figure 5:**
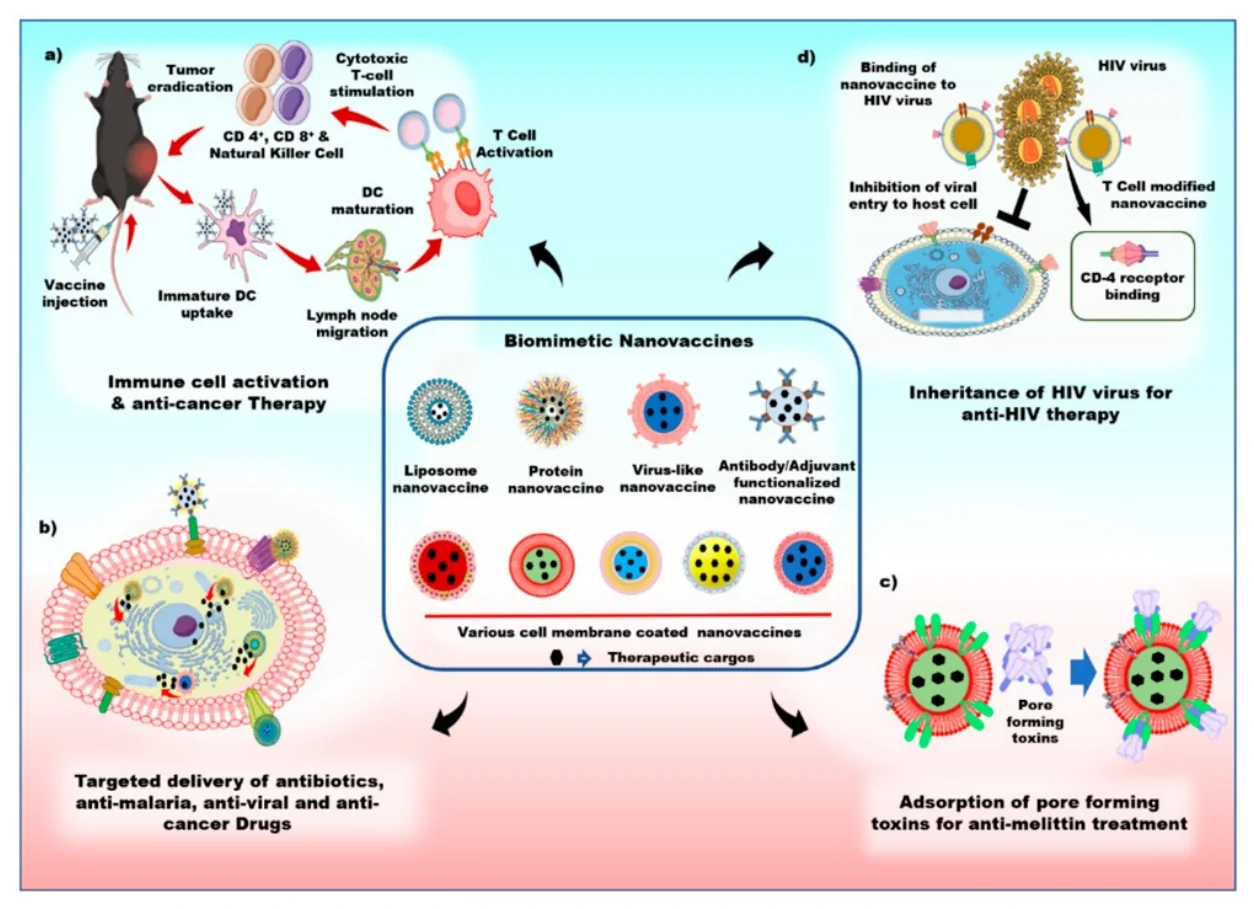
Schematic representation of the therapeutic applications of biomimetic nanovaccines, including immune activation, targeted drug delivery, toxin neutralization and viral inhibition. (**a**) Immune cell activation for tumor eradication; (**b**) targeted drug delivery; (**c**) pore-forming toxin neutralization; (**d**) viral entry inhibition via CD4 receptor binding. Reproduced from Ref. [192].

Cancer cell membrane-based nanovaccines provide a broad repertoire of tumor-associated antigens (TAAs) while offering excellent biocompatibility and potential for multivalent and personalized immunotherapy [191]. Figure 5 illustrates applications of biomimetic nanovaccines across membrane types and should not be interpreted as evidence that cancer-cell membranes provide toxin neutralization or antiviral protection. Native tumor membranes may require antigen enrichment or adjuvants to induce effective antigen-presenting-cell activation and antitumor immunity [192]. Engineering membranes with immunogenic cell death (ICD)-associated signals, such as enhanced calreticulin expression, can improve antigen-presenting cell uptake and activation, thereby strengthening antigen presentation and antitumor immune responses [193]. A senescent cancer cell membrane-coated mesoporous silica nanoadjuvant loaded with CpG (SCCM@NA) was developed as a biomimetic platform for melanoma immunotherapy [194]. The biomimetic platform enhanced dendritic cell uptake and lymph-node targeting, promoted CD8^+^ T-cell activation, and synergized with anti-PD-1 therapy to enhance antitumour immunity and suppress metastasis. Collectively, these studies highlight the potential of CCM-NPs for targeted drug delivery and cancer immunotherapy, although their targeting, immune interactions, and therapeutic performance depend on the cancer cell membrane source, nanoparticle formulation, and tumor model.

Immune evasion and vaccination impose different design requirements: reduced phagocytic uptake may favor systemic drug delivery, whereas vaccines require efficient uptake or antigen transfer to antigen-presenting cells. Potentially immunosuppressive membrane components should therefore be assessed for their effects on vaccine potency. Compared with bacterial membranes, cancer-cell membranes provide tumor-derived antigen information but generally require deliberate control of adjuvant activity. For example, antigen-enriched tumor membranes combined with PC7A induced source-specific antitumor responses in murine and humanized models, with peptide–MHC-I transfer to dendritic cells supporting cross-dressing [195]. These findings are preclinical and do not establish that all native tumor membranes are effective vaccines.

### 8.3. Photothermal Therapy

CCM-NPs have been investigated for tumor-targeted delivery combined with photothermal therapy, chemotherapy, and immunomodulation. In a CT26 tumor model, cancer cell membrane-coated bismuth nanoparticles (Bi@CCM NPs) retained the photothermal activity of the Bi core. Following intravenous administration, the coated formulation was reported to have an approximately 2.9-fold longer circulation half-life and 2.3-fold greater tumor accumulation than uncoated Bi NPs. With 808 nm laser irradiation, Bi@CCM NPs produced near-complete tumor eradication within 12 days under the tested conditions [196]. Cancer cell membrane-coated palbociclib-loaded mesoporous silica–polypyrrole nanoparticles (CM@SP-PLB NPs) were developed as a biomimetic platform for homotypic targeting and combined chemo-photothermal immunomodulatory therapy of oral cancer [197]. The nanoparticles exhibited laser-responsive drug release, a photothermal conversion efficiency of 57.6%, and enhanced homotypic uptake by oral cancer cells. Treatment induced ROS generation, mitochondrial dysfunction, and immunogenic cell death, as indicated by calreticulin exposure and extracellular ATP release. In vivo, CM@SP-PLB NPs showed enhanced tumour accumulation and, following laser irradiation, increased tumour temperature to approximately 55 °C, resulting in substantial tumour regression. The treatment also promoted tumour-associated macrophage repolarization and antitumour immune activation, while demonstrating favourable systemic biocompatibility and reduced metastasis. These findings describe the combined formulation and treatment: photothermal conversion and drug effects arise from the core and cargo, while the membrane contribution requires matched uncoated and alternative-membrane controls. Favorable short-term tolerability does not establish repeated-dose or long-term safety. The cancer-cell source is most directly relevant to oncology; extensions to other diseases require a separate targeting and safety rationale. Presently, CCM-NPs have predominantly been investigated in oncology, their potential applications in non-cancer diseases remain comparatively underexplored. Selected cancer cell membrane-coated nanoplatforms are compared by cargo/core, cancer application, experimental model, administration regimen, comparators, outcomes, and evidence limitations, as summarized in Table 6.

### 8.4. Safety Scalability and Potential Clinical Use

Cancer-cell membranes (CCMs) raise safety concerns beyond RBC or platelet membranes. Although isolated membranes cannot replicate, manufacturing must exclude viable tumor cells and control residual DNA, intracellular material, contaminants, and potentially tumor-promoting or immunosuppressive components. Purification should be linked to functional testing because it may alter adhesion molecules or tumor antigens [176,202]. Autologous membranes may improve antigen matching but do not guarantee safety, while allogeneic sources require assessment of immune incompatibility. Biodistribution, clearance, repeated-dose immunogenicity, and core- and cargo-related toxicity also require evaluation [202,203]. Established cancer-cell lines enable cell banking and expansion but may undergo culture- or passage-related phenotypic changes, requiring batch control of identity and membrane potency. Patient-derived membranes offer closer tumor matching but face limitations in tissue availability, heterogeneity, processing time, and scalability. Production must also control coating completeness, membrane-to-core ratio, residual free membrane, sterility, and stability. Potential clinical applications include source-matched tumor targeting and tumor-antigen vaccination [176]. Selection of CCMs over RBC, platelet, stem-cell, immune-cell, or bacterial membranes should therefore depend on the intended function and comparative evidence. Translation requires reproducible source qualification, validated safety and potency assays, scalable manufacturing, and evidence that the membrane provides measurable benefit over appropriate controls [202,203].

## 9. Patents and Clinical Trials

Recent patent activity highlights growing commercial interest in cell membrane-coated nanoparticles (CM-NPs) for diverse biomedical applications, particularly in cancer therapy and infectious diseases. A structured patent search was conducted to identify recent intellectual property related to cell membrane-coated nanoparticle technologies. Relevant patent databases, including Google Patents, WIPO PATENTSCOPE, and Espacenet, were searched using combinations of terms such as “cell membrane-coated nanoparticles,” “biomimetic nanoparticles,” “membrane-coated nanocarriers,” and specific membrane sources. Patents were selected based on their relevance to CM-NP fabrication, functionalization, drug delivery, therapeutic applications, and clinical translation. The identified patents are summarized in the corresponding table to illustrate emerging innovation and commercialization trends in CM-NP technology. The retained patents encompass platelet–neutrophil hybrid membranes, tumor cell membranes, bacterial membranes, and bacterial outer membrane vesicles applied to engineered cores. These identified patents encompass diverse membrane sources, including platelet–neutrophil hybrid membranes, tumor cell membranes, bacterial membranes, and bacterial outer membrane vesicles used to coat synthetic nanoparticle cores. These patented technologies combine membrane-derived biological properties with engineered nanoparticle cores for applications including chemotherapy, photothermal and photodynamic therapy, MRI-guided drug delivery, targeted protein degradation, antibacterial immunotherapy, and multimodal cancer therapy. Collectively, these patents reflect growing translational interest in CM-NPs and efforts to advance these biomimetic nanoplatforms toward biomedical applications. Table 7 summarizes recent patents on CM-NPs, highlighting their membrane sources, therapeutic applications, and key inventions.

Despite substantial progress in nanoparticles coated with membranes derived from RBCs, platelets, cancer cells, stem cells, immune cells, bacteria, and hybrid membrane systems, their clinical translation remains at an early stage. Preclinical studies have demonstrated reduced immune clearance, prolonged systemic circulation, enhanced disease-specific targeting, and multimodal therapeutic activity in specific CM-NP formulations; however, these outcomes vary depending on the membrane source, nanoparticle composition, fabrication method, and experimental model. To date, the vast majority of CM-NP formulations remain at the preclinical stage, with very few platforms progressing toward human clinical evaluation. Patents in Table 7 reflect intellectual property activity, not demonstrated clinical readiness. Translation remains limited by membrane variability, inconsistent coating coverage and protein orientation, GMP scale-up, and validated quality and potency assays. Size shifts and protein detection alone cannot confirm complete coating or retained function. Further barriers include residual cellular material and free vesicles, sterility and endotoxin control, storage stability, and formulation-specific safety. Biodistribution, clearance, repeated-dose immunogenicity, and source-specific risks require assessment. These requirements increase costs and regulatory complexity, while comparisons with uncoated cores and standard treatments are needed to establish membrane-related benefits. Top-down CM-NPs comprise isolated cell membranes applied to independently fabricated cores; drug-loaded EVs and EV-coated cores are distinct platforms. NCT01854866 and NCT02657460 evaluated tumor-derived microparticles loaded with chemotherapeutic agents for malignant ascites and pleural effusion and therefore do not directly validate synthetic-core CM-NPs. More recently, Cello Therapeutics received U.S. FDA Investigational New Drug (IND) clearance in 2023 for CE120, a platelet membrane-based nanotherapeutic platform, representing progress toward clinical evaluation of membrane-based nanotechnology. The approval permits clinical investigation but does not establish efficacy or marketing approval. Clinical translation requires reproducible manufacturing, validated quality and potency assays, formulation-specific safety data, and controlled human studies.

## 10. Future Perspectives and Challenges

Cell membrane-coated nanoparticles (CM-NPs) integrate biological membranes with synthetic nanoparticle cores and have demonstrated potential for improving circulation, immune interactions, targeted delivery, and therapeutic efficacy in preclinical studies. However, these benefits are not inherent to membrane coating and depend on factors such as the membrane source, nanoparticle composition, coating integrity, fabrication method, and biological context. A major challenge is the lack of standardized protocols for membrane isolation, purification, characterization, storage, and coating, resulting in batch variability and inconsistent preservation and orientation of membrane components that are critical for biological functionality [2,209,210]. Furthermore, heterogeneity among membrane sources and donor-to-donor variability complicates reproducibility, standardization, and quality assurance across manufacturing batches.

Importantly, translational barriers are membrane-source dependent. RBC and platelet membranes require donor qualification, compatibility, and pathogen control; cancer-cell membranes raise concerns regarding tumor-derived components and residual cellular material; bacterial membranes require stringent control of endotoxins, immunogenicity, and reactogenicity; while stem-cell membranes require control of donor, culture, and phenotypic variability. These source-specific differences may also influence scalability, reproducibility, safety assessment, and regulatory requirements.

Large-scale manufacturing represents another major obstacle. Current preparation methods—including membrane extraction, vesicle formation, extrusion, sonication, electroporation, and membrane–nanoparticle fusion—can be labor-intensive, difficult to automate, and associated with variable production yields. Future research should focus on scalable, good manufacturing practices (GMP)-compliant manufacturing with standardized production, purification, and quality control to ensure reproducibility and support the clinical translation of cell membrane-coated nanoparticles [73,210,211]. Standardized quality control must extend beyond particle size, zeta potential, drug loading, and storage stability to verify membrane orientation, coating completeness, marker accessibility, and biological potency. As detailed in Section 3, complementary analyses include domain-specific antibody assays for orientation, microscopy combined with quantitative core-accessibility assays for coating integrity, and protein profiling with calibrated immunodetection for surface markers [74,75,76,77]. FRET can support hybrid membrane mixing but cannot independently establish complete coating [66]. These attributes should be linked to mechanism-specific functional assays and blocking controls, alongside sterility, endotoxin, residual nucleic acid, microbial contamination, and stability testing.

Sterility assurance presents unique challenges because membrane-coated nanoparticles are biological–synthetic hybrid systems that cannot always withstand conventional terminal sterilization methods such as steam sterilization or gamma irradiation without compromising membrane integrity and biological functionality. Therefore, aseptic processing, validated clean-room manufacturing, sterile filtration of individual components where technically feasible, and validated sterility testing strategies are important considerations for clinical production. In addition, stringent evaluation of pyrogenicity, endotoxin contamination (particularly for bacterial membrane-derived systems), viral safety, mycoplasma contamination, and immunogenicity should be incorporated into manufacturing and quality-control workflows to ensure product safety [210,212,213].

In BCM-NPs, LPS may be intrinsic to the membrane or introduced during processing. Parenteral endotoxin limits are calculated as L = K/M, with K generally 5 EU/kg for non-intrathecal administration and 0.2 EU/kg for intrathecal administration, and M representing the maximum human dose per kilogram within one hour. A non-intrathecal dose of 10 mg/kg yields a limit of 0.5 EU/mg. Applicable monograph or product-specific requirements take precedence [214]. Final formulations require endotoxin testing under USP <85> or validated recombinant methods under USP <86>, which provides alternative assays rather than separate safety limits. Method suitability must confirm spike recovery and exclude interference or masking within the maximum valid dilution; a low signal alone does not demonstrate removal. Testing should also address non-endotoxin pyrogens and formulation-specific inflammatory responses [214,215]. Deleting lipid A acyltransferase genes, such as msbB, can reduce acylation and endotoxicity without eliminating LPS, as demonstrated in Vibrio cholerae OMVs [216]. Source engineering should be followed by membrane purification, core coating, and final-product testing. OMV findings do not establish BCM-NP compliance; lipid A modification, marker retention, coating integrity, potency, and residual endotoxin require formulation-specific verification. Where compatible, immobilized polymyxin B can remove accessible LPS from soluble process fractions, but selective removal of membrane-integrated LPS from intact BCM-NPs remains unverified [217]. Processing must assess endotoxin reduction, membrane or particle recovery, residual polymyxin B, and retained function. Binding or reduced cytokine responses alone cannot establish compliance; validated final-product endotoxin testing is required.

From a regulatory perspective, membrane-coated nanoparticles represent a new class of complex biological nanomedicines that do not fit neatly within existing regulatory frameworks for conventional pharmaceuticals, biologics, and established nanomedicines. Although existing regulatory principles for nanomedicines and biological products provide relevant guidance, specific regulatory standards tailored to cell membrane-coated nanoparticles remain limited, particularly regarding critical quality attributes, comparability following manufacturing changes, potency assays, and long-term safety evaluation. Future regulatory approaches for cell membrane-coated nanotherapeutics will likely require comprehensive physicochemical characterization, pharmacokinetic and biodistribution profiling, immunotoxicity assessment, degradation and clearance studies, as well as product-specific evaluation of other relevant safety considerations. Standardized analytical methods and reference standards will also be important for ensuring product quality, safety, and reproducibility [218,219,220]. Experience gained from liposomal formulations, antibody-drug conjugates, biologics, and other nanomedicines may provide valuable regulatory precedents, although CM-NPs introduce additional complexities arising from their biological membrane components.

Future research should also emphasize membrane engineering through genetic modification, metabolic engineering, click chemistry, and synthetic biology to enhance targeting specificity, reduce immunogenicity, and introduce programmable therapeutic functions. Integration of artificial intelligence and machine learning with high-throughput screening, computational modeling, and advanced manufacturing approaches may accelerate the optimization of membrane composition, nanoparticle design, and therapeutic performance. Furthermore, hybrid membrane systems combining membranes from multiple cell sources may provide complementary biological functions and help overcome some of the limitations associated with single-source membrane coatings. Beyond oncology, expanding applications to cardiovascular, neurological, autoimmune, infectious, metabolic, and regenerative diseases will further broaden the therapeutic potential of these biomimetic platforms. Ultimately, multidisciplinary collaboration, harmonized regulatory standards, and well-designed clinical trials will be essential for translating cell membrane-coated drug delivery systems from preclinical research toward clinically translatable nanomedicines.

## 11. Conclusions

Biomimetic cell membrane-coated nanoparticles (CM-NPs) integrate natural membrane components with tunable nanocarrier properties and have demonstrated potential to improve biocompatibility, modulate immune recognition, prolong circulation, and facilitate targeted therapeutic delivery. However, these outcomes depend on the membrane source, nanoparticle composition, coating integrity, fabrication method, and biological context. Different membrane sources provide distinct biological properties that can be exploited for specific therapeutic applications. CM-NPs have been extensively investigated in cancer therapy for targeted drug delivery, immune modulation, photothermal and photodynamic therapy, and combination treatment strategies, with emerging applications in cardiovascular, neurological, infectious, inflammatory, and regenerative diseases. Despite promising preclinical findings, clinical translation remains limited by variability in membrane sources and coating processes, manufacturing scalability, stability, quality control, biosafety, and regulatory challenges. Cancer cell membrane-coated nanoparticles present additional concerns related to membrane purity, removal of potentially tumor-promoting components, source-cell availability, patient acceptance, personalized production, batch consistency, and scale-up. Future research should prioritize standardized and scalable fabrication, rigorous characterization, reproducible quality control, and comprehensive evaluation of pharmacokinetics, biodistribution, immunogenicity, toxicity, and therapeutic efficacy. Advances in membrane engineering, hybrid membrane systems, stimuli-responsive designs, and theranostic platforms, together with scalable manufacturing and regulatory standardization, may facilitate the development of safe, reproducible, and clinically translatable CM-NPs.

## Figures and Tables

**Table 1:** Surface engineering strategies for cell membrane-coated nanoparticles.

Strategy	Principle	Typical Modification/Approach	Key Advantages	Major Limitations	Representative Applications
Lipid insertion	Hydrophobic insertion of amphiphilic anchors into the membrane bilayer	Cholesterol, DSPE/DSPE–PEG derivatives	Simple, rapid, minimally invasive; preserves membrane proteins	Anchor desorption/exchange; transient retention	Targeting and immunomodulatory ligand display
Covalent conjugation	Formation of stable bonds with membrane functional groups	EDC/NHS-thiol, click chemistry	Stable ligand attachment; controllable functionalization	May alter protein conformation or ligand orientation	Antibody- and peptide-mediated targeting
Metabolic glycoengineering	Biosynthetic incorporation of bioorthogonal groups into cell-surface glycans	Azidosugars followed SPAAC	High specificity; minimal membrane perturbation	Requires viable donor cells; longer processing	Targeted delivery and bioorthogonal labeling
Genetic engineering	Modification of donor cells to express functional membrane proteins	CRISPR/Cas9, transfection, engineered receptors	Native protein orientation; precise and reproducible expression	Cost, manufacturing complexity, biosafety and regulatory concerns	CXCR4-, PD-1-, CD47-, and SpyCatcher-engineered membranes

**Table 2:** Comparison of membrane-coating techniques in terms of coating quality, membrane preservation, batch uniformity, material yield, throughput and batch capacity, and potential for GMP scale-up.

Technique	Coating Efficiency	Membrane Integrity and Protein Orientation	Batch Uniformity	Material Yield	Throughput	Scalable GMP Feasibility	References
Extrusion	Promotes membrane–core fusion; full coating fraction not quantified.	Repeated mechanical stress may affect membranes and proteins; orientation requires verification.	Consistent particle sizes; coating heterogeneity requires separate assessment.	Formulation specific	Constrained by repeated passes and clogging	Scale-up limited by clogging and material loss; requires validated process controls.	[72,73]
Sonication	Facilitates fusion; efficiency depends on optimized energy and exposure.	Excess energy may disrupt membranes or denature proteins; orientation unverified.	Excess sonication may increase size heterogeneity.	Potentially fewer handling losses; Recovery not reported	Depend on sample volume and energy delivery.	Requires control of energy exposure, heating, and batch consistency; GMP scalability not established.	[66,68]
Electroporation	Transient permeabilization supports association; efficiency not quantified.	Electrical stress may affect integrity and protein function; orientation requires verification.	Controlled assembly possible	Recovery not reported	Depends on process configuration	Requires optimized electrical parameters and validated reproducibility; GMP feasibility remains unestablished.	[69,71]
In situ polymerization	Core formation within or near vesicles may improve coating; efficiency not quantified.	Reaction conditions and residual reagents may affect membrane components; orientation unverified.	Depends on polymerization control	Recovery not reported	Depends on reaction and purification;	Requires reaction control, residual-reagent removal, and membrane-function validation.	[70]
Microfluidic coating	Controlled mixing supports consistent coating; full coverage requires verification.	Shear may affect protein integrity; orientation requires independent assessment.	More uniform sizes and improved process consistency described.	Recovery not reported	Controlled processing offers scale-up potential;	Promising process control and scalability; specialized equipment and GMP validation required.	[69,71]

**Table 3:** Representative RBC membrane-coated nanoplatforms with experimental models, administration routes, comparators, quantitative outcomes and evidence limitations.

Nanoplatform and Cargo/Core	Application	Experimental Model	Route and Regimen	Comparators and Uncoated Core	Reported Outcomes	Evidence level and Membrane Contribution	References
RBC membrane-coated PLGA NPs loaded with DOX [RBC-NP(DOX)]	Solid lymphoma chemotherapy	In vitro: EL4 murine lymphoma cells. In vivo: EL4 tumor-bearing male C57BL/6 mice	IV tail vein; 3 mg/kg DOX, every other day for 2 weeks	Free DOX; empty RBC-NP; isotonic sucrose. Uncoated DOX-loaded PLGA core not included	Median survival increased to 47 vs. 24 days, with no observable myelosuppression or anti-RBC IgM/IgG response.	Preclinical only (*n* = 5 efficacy; *n* = 3 safety); no uncoated PLGA comparator, limiting membrane-specific attribution.	[101]
Fe_3_O_4_@RBC nanoparticles RBC membrane-coated iron oxide core	Prolonged circulation and reduced accelerated blood clearance	In vitro: RAW 264.7 murine macrophages. In vivo: male ICR mice, 6–8 weeks	Tail-vein injection; 25 mg Fe/kg; repeated dose after 5 days for ABC assessment	PBS; uncoated Fe_3_O_4_ NPs; Fe_3_O_4_@PEG NPs	RBC coating reduced macrophage uptake, prolonged circulation (14.2% ID/g at 24 h), reduced organ accumulation, and prevented ABC and IgM/IgG elevation.	Preclinical study *(n* = 4/group) with uncoated and PEG controls; long-term safety remains unestablished.	[67]
RBC membrane-coated PLGA nanosponges	Neutralization/detoxification of pore-forming toxins	In vitro: mouse RBCs, HUVECs. In vivo: nude/ICR mice challenged with α-toxin	SC toxin-neutralization model; IV systemic model: 80 mg/kg nanosponge, α-toxin 75 μg/kg	PEG-PLGA NPs; RBC membrane vesicles; PEG-liposomes (in vitro); untreated control	Bound 90.2% α-toxin; nanosponge pretreatment reduced mortality from 100% to 11%; post-treatment reduced mortality to 56%.	Preclinical only (*n* = 9 systemic survival); PEG-PLGA and RBC-vesicle controls included, but no uncoated PLGA-core control.	[102]
RBC membrane-coated chitosan NPs	Detoxification of *E. coli*- and *Salmonella typhi*-derived LPS; attenuation of inflammation and oxidative stress	In vivo: albino rats, 250–300 g; *n* = 5/group, 7 groups (35 rats)	SC; LPS 150 µL/kg; RBC-coated NPs 20 µg/kg; treatment on day 3 after LPS challenge	Healthy control; LPS-only controls; PEG-coated chitosan NPs	RBC-coated NPs reduced fever (102.2 → 98.7 °F), WBCs (17.5 → 11.7 × 10^3^/cm), and TNF-α (3.14 → 1.20 pg/mL) and restored CAT/SOD toward normal levels.	Single small-animal study (*n* = 5/group); no uncoated chitosan NP treatment group, limiting membrane-specific attribution.	[103]
RBC membrane-coated PLGA/AIEgen/Poly(I:C) NPs (M@AP)	Photodynamic-immunotherapy of melanoma and lung metastasis	In vitro: B16-F10 melanoma cells, RAW264.7, PBMCs. In vivo: B16-F10 tumor-bearing C57BL/6 mice	IV for PK/biodistribution; intratumoral administration + light irradiation for bilateral tumor model	PBS; M@A; M@AP; M@A + light; uncoated AP included for PK/biodistribution	RBC coating prolonged circulation and tumor/spleen accumulation; M@AP + light suppressed primary/distant tumors and lung metastasis and increased CD4+/CD8+ T cells.	Preclinical (*n* = 5 efficacy; *n* = 3 immune analyses); no uncoated AP efficacy control, limiting membrane-specific attribution.	[104]
RBC membrane-coated FePt@ZIF-8 (FePt@ZIF-m)	MRI-guided magnetic hyperthermia for HCC	In vitro: SK-HEP-1 and Mahlavu HCC cells. In vivo: orthotopic HCC mouse model	IV, 100 μL of 5 mg/mL FePt@ZIF-m + magnetic hyperthermia	PBS control; uncoated FePt@ZIF used in physicochemical/in vitro studies, but not as in vivo efficacy control	FePt@ZIF-m enhanced MRI contrast, inhibited tumor growth, and improved survival without significant weight changes.	Preclinical (*n* = 5); no uncoated FePt@ZIF efficacy control, limiting membrane-specific attribution.	[105]
RBC membrane-coated supramolecular gelatin NPs loaded with vancomycin + chlorogenic acid (VC-SGNPs-R)	MRSA/MDR infection; toxin-neutralizing, infection-responsive antibiotic delivery	In vitro: MRSA USA300; RAW264.7 macrophages. In vivo: male SD mice with leg and skin MRSA infection models	IV tail vein; 100 μL, 2 mg/kg; treatment over 5 days	PBS, SGNPs, free VC, uncoated VC-SGNPs, SGNPs-R	RBC coating reduced macrophage uptake; ~85% drug release at 6 h was achieved, with reduced bacterial burden, inflammation, and wound size without weight loss.	Preclinical (*n* = 4/group); uncoated comparator included, supporting membrane-specific immune-evasion effects, but in vitro antibacterial efficacy was similar.	[106]

**Table 4:** Selected studies of platelet membrane-coated nanoplatforms, highlighting their therapeutic cargo/core, disease applications, and major therapeutic outcomes.

Nanoplatform and Cargo/Core	Application	Experimental Model	Route and Regimen	Comparators and Uncoated Core	Reported Outcomes	Evidence Level and Membrane Contribution	References
rVAR2-PM/PLGA-ss-HA: DTX-loaded, disulfide-linked PLGA–HA nanoparticles with rVAR2-modified platelet coating.	Primary melanoma and lung metastases	In vitro: B16F10 cells and RAW264.7 macrophages. In vivo: B16F10 tumor and lung metastasis models in C57BL/6 mice.	IV DTX, 4 mg/kg/day for ~1 week.	PBS; free DTX; uncoated PLGA-ss-HA; PM-coated core without rVAR2.	Enhanced tumor uptake and reduced macrophage uptake; apoptosis 56.53% vs. 40.29% with uncoated core; lung nodules 21.0 ± 5.39 vs. 78.2 ± 6.61 with PBS; reduced tumor growth without weight loss.	Single preclinical study (*n* = 5/group efficacy). Uncoated and PM-only controls support coating and rVAR2 effects; no rVAR2-modified uncoated control or survival endpoint.	[129]
H-L-D-Z@PM: platelet membrane-coated ZIF8 containing hemoglobin, lactate oxidase and DOX (~964 nm).	Breast cancer chemotherapy; relief of tumor acidosis and hypoxia.	In vitro: 4T1 cells and HUVECs. In vivo: Subcutaneous 4T1 tumors in female BALB/c mice.	IV, 200 μL of 2 mg/mL nanoparticles, every 3 days × 3; 14-day assessment.	PBS; PM-coated ZIF8 with individual/combined cargo. Uncoated H-L-D-Z evaluated for cytotoxicity and biodistribution, but not therapeutic efficacy.	Tumor fluorescence peaked at 6 h (uncoated: 12 h); reduced lactate, hypoxia and tumor growth without weight loss; HUVEC viability > 80% at 200 μg/mL.	Preclinical (*n* = 5/group); coating improved tumor accumulation and compatibility, but no uncoated efficacy control confirmed membrane-specific benefit.	[130]
LPHM@DDI: DOX/IR780-loaded dendritic large-pore mesoporous silica with leukocyte/platelet hybrid membrane	Triple-negative breast cancer; chemo/PTT/PDT combination.	In vitro: 4T1 cells; RAW264.7 macrophages. In vivo: subcutaneous 4T1 tumors in female BALB/c mice	Single IV dose: DOX 1.5 mg/kg, IR780 2 mg/kg; 808 nm laser, 2 W/cm^2^, 12 h later for 5 min (efficacy Results); 21-day follow-up.	PBS; free DOX/IR780 mixture; uncoated DDI; leukocyte-only LM@DDI; platelet-only PM@DDI; treatments with/without laser.	Reduced macrophage uptake and increased tumor accumulation; laser heating > 50 °C suppressed tumor growth, recurrence and angiogenesis without significant weight change.	Single preclinical study; group sizes not specified in main text. Uncoated and single-membrane controls support hybrid-coating benefits in vivo; 4T1 uptake was similar to platelet-only coating.	[131]
pH-HCM@FeCNDs: iron–gallic acid nanodots in a platelet membrane/pH-responsive liposome hybrid.	Breast cancer; combined PTT/CDT; lung metastasis suppression.	In vitro: 4T1 cells and multicellular spheroids. In vivo: orthotopic 4T1 breast tumors with lung metastases in BALB/c mice.	IV, 3 mg Fe/kg; 808 nm laser, 1.2 W/cm^2^ for 6 min, 24 h later; ~25-day follow-up.	PBS; laser alone; formulation without laser; uncoated FeCNDs + laser; pH-responsive liposomes + laser; nonresponsive platelet hybrid + laser.	With laser: 90.33% tumor reduction versus PBS; lung nodules 1.25 versus 17.8 (PBS); lung tumor coverage 0.29% versus 8.22%. Three of five primary tumors eradicated without recurrence during follow-up.	Single preclinical study (*n* = 5/group efficacy; *n* = 3 imaging/safety). Uncoated, liposome-only and nonresponsive hybrid controls support platelet targeting and pH-responsive release; short follow-up.	[132]
TRAIL-Dox-PM-NV: platelet-coated acid-degradable nanogel with surface TRAIL and core DOX (~121 nm).	Breast cancer; sequential drug delivery and suppression of circulating tumor cell-mediated lung metastasis.	In vitro: MDA-MB-231 cells; RAW264.7 macrophages. In vivo: MDA-MB-231 tumors and IV tumor-cell lung colonization in nude mice.	IV; TRAIL 1 mg/kg and DOX 2 mg/kg. Tumors assessed on day 16; lung metastases at 8 weeks. Dosing frequency not specified in main text.	Saline; uncoated TRAIL-Dox-NV; TRAIL-PM-NV; Dox-PM-NV. Metastasis: saline, uncoated dual-drug NV and coated dual-drug nanovesicle.	Tumor fluorescence 1.9-fold higher than uncoated NV; reduced macrophage uptake; strongest tumor inhibition and fewer lung nodules versus uncoated dual-drug NV (*p* < 0.001), with stable body weight.	Single preclinical study (*n* = 5 tumor efficacy; *n* = 3 metastasis). Uncoated dual-drug control supports membrane contribution; lung model tests experimentally introduced circulating cells, not spontaneous metastasis.	[133]
FK506-PNPs: tacrolimus-loaded PLGA nanoparticles coated with platelet membrane (~122 nm).	Targeted rheumatoid arthritis treatment.	In vitro: MH7A synoviocytes on collagen IV-coated dishes. In vivo: collagen-induced arthritis in DBA/1 mice; ICR mice for circulation/safety studies.	IV FK506, 1 mg/kg every other day for 2 weeks.	Saline/PBS; free FK506; uncoated FK506-PLGA NPs; healthy mice. Imaging: free dye, uncoated and coated NPs.	Joint accumulation 2.1-fold higher than uncoated NPs at 24 h; prolonged blood retention. Lowest arthritis index, reduced inflammatory cytokines, and less bone/cartilage damage versus free drug and uncoated NPs.	Single preclinical study (*n* = 5/group efficacy; *n* = 3 imaging). Uncoated drug-loaded controls support coating benefit; individual receptor contributions were not isolated.	[134]
PLT-MNs: autologous platelet membrane-coated Fe_3_O_4_ magnetic nanoparticles (~100 nm); no drug cargo.	Breast tumor MRI and PTT.	In vitro: MCF-7 cells; RAW264.7 macrophages. In vivo: MCF-7 xenografts in BALB/c nude mice; ICR mice for safety and immune response.	IV, 2.5 mg Fe/kg; 808 nm laser, 5 W/cm^2^ for 5 min, 1 h later; with/without external magnetic field (MF).	PBS; uncoated MNs; RBC-coated MNs; PLT-MNs with/without laser or MF.	Reduced macrophage uptake; enhanced tumor accumulation and T2 MRI contrast; laser heating to 56.1 °C (MNs: 49.5 °C; RBC-MNs: 53.6 °C). PLT-MNs + MF + laser achieved near-complete ablation without significant weight change.	Preclinical (*n* = 6/group); uncoated/RBC-coated controls support platelet targeting; MF enhanced PTT. Follow-up inconsistent: 16 vs. 30 days.	[135]
PLT@Ag-MOF-Vanc: platelet membrane-coated silver MOF loaded with vancomycin (~148 nm).	MRSA pneumonia; targeted antibacterial and antibiofilm therapy.	In vitro: MRSA cultures/biofilms; *E. coli*, *P. aeruginosa* and *S. aureus*; RAW264.7 cells for compatibility. In vivo: MRSA pneumonia in male Kunming mice.	IV tail vein; vancomycin 2 mg/kg/day × 3, starting 8 h after intratracheal MRSA challenge; survival monitored for 7 days.	Saline; Ag-MOF; free vancomycin; uncoated Ag-MOF-Vanc. Ag-MOF dose matched to formulation content.	Enhanced lung targeting; reduced bacteria, biofilms and inflammation; 7-day survival 100% vs. 50% with free vancomycin (2 mg/kg), without obvious short-term toxicity.	Preclinical (*n* = 5 efficacy; *n* = 10 survival/group); uncoated control supports membrane-related targeting and efficacy; short follow-up.	[136]

**Table 5:** Representative bacterial membrane-coated nanoparticles (BCM-NPs), including cargo/core, applications, experimental models, administration regimens, comparators, reported outcomes, and evidence limitations.

Nanoplatform and Cargo/Core	Application	Experimental Model In Vitro and In Vivo	Route and Regimen	Comparators and Uncoated Core	Reported Outcomes	Evidence Level and Membrane Contribution	References
IR780@PLGA@HM: IR780-loaded PLGA coated with *E. coli* DH5α OMV–4T1 cell hybrid membrane (1:1; ~200–215 nm).	Breast cancer bone lesions; SDT and immune modulation.	In vitro: 4T1, RAW264.7, L929 cells; primary BMDMs. In vivo: intratibial 4T1 tumors in female BALB/c mice.	IV 200 µL, 10 mg/mL nanoparticles, twice weekly for 2 weeks; US 24 h later: 1 MHz, 1 W/cm^2^, 10 s on/off for 2 min.	PBS; US alone; uncoated IR780@PLGA; 4T1-only-coated IR780@PLGA@4T1; hybrid-coated particles; each formulation ±US.	Enhanced tumor accumulation; M1 polarization and DC activation. Hybrid +US reduced tumor growth and bone damage; 14-day survival 100% vs. 50% with PBS, with no significant weight difference.	Preclinical (*n* = 10/group efficacy; *n* = 3 imaging/safety). Uncoated and 4T1-only controls support coating benefits; no OMV-only coating control. Direct bone implantation; short follow-up.	[167]
BNP: PC7A/CpG polyplex coated with Mycobacterium smegmatis membrane and surface maleimide groups (~207 nm).	In situ vaccination with RT.	In vitro: DCs with ovalbumin or B78 tumor lysate. In vivo: B78 melanoma in C57BL/6 mice; NXS2 neuroblastoma in A/J mice; tumor rechallenge.	Intratumoral BNP on days 1, 3, 5, 7 and 9; tumor RT 12 Gy on day 1. Dose unspecified in attachment; survival to day 60; rechallenge at day 90.	PBS; RT alone; BNP alone; RT +BNP; BNP without maleimide; membrane, PC7A, CpG and uncoated PC7A +CpG component controls with RT.	Enhanced antigen capture, DC uptake/activation and endosomal escape. RT +BNP: 100% B78 response; 100% NXS2 primary regression but 50% survival. Rechallenge rejection: 100% B78, 75% NXS2.	Single preclinical study (pooled *n* = 12/group B78; *n* = 8 NXS2; *n* = 10 component study). Controls support the full formulation; membrane effects not fully isolated. Late tumor/metastatic relapse occurred.	[168]
OM-NPs: PLGA cores coated with H. pylori outer membrane (~106 nm); no drug cargo.	H. pylori anti-adhesion; bacterial decoys.	In vitro: H. pylori adhesion to AGS gastric epithelial cells. Ex vivo: freshly excised mouse stomach tissue. In vivo: not evaluated.	Direct incubation; pretreatment or simultaneous bacterial exposure. Ex vivo: 40 mg/mL OM-NPs for 1.5 h before H. pylori (5 × 10^7^ CFU/mL).	PBS/no OM-NPs; anti-H. pylori antibody-blocked OM-NPs for cell binding. Bare PLGA/OMVs in characterization; no uncoated core anti-adhesion control.	Adhesion IC50: 0.034 mg/mL pretreatment vs. 0.23 mg/mL simultaneous dosing. At 40 mg/mL, 35.7% bacteria remained on AGS cells at 22 h. Ex vivo adhesion: 46.2 ± 13.1% of PBS control.	In vitro/ex vivo only (*n* = 3 cell assays; *n* = 6 tissue assay). Antibody blocking supports membrane-mediated binding; no uncoated efficacy control or live-animal infection study.	[171]
CpG@MSN-PEG/PEI@OMVs: CpG-loaded PEG/PEI-modified mesoporous silica coated with *E. coli* BL21 (DE3) ΔLpp OMVs (~69 nm).	Cancer immune therapy; T-cell restoration and metastasis inhibition.	In vitro: RAW264.7 cells; BMDCs. In vivo: 4T1 tumors/lung metastases (female BALB/c); B16F10 lung metastases and MC38 tumors/rechallenge (male C57BL/6).	IV. 4T1: days 13, 16; CpG 8.6 µg/mouse, OMV protein 8 µg/mouse. MC38: days 5, 7, 9, 11, 13; ±IP anti-PD-L1 200 µg/dose.	PBS; empty uncoated MSN-PEG/PEI (200 mg/kg); OMV-coated core without CpG; CpG-loaded coated core. MC38: ±anti-PD-L1. No CpG-loaded uncoated efficacy control.	Enhanced tumor targeting and immune activation; reduced lung metastases and 4T1 tumor weight (~3.2-fold vs. PBS). MC38 +anti-PD-L1: 83.3% remission, 60% rechallenge protection; stable body weight.	Preclinical (*n* = 6 efficacy; *n* = 4 distribution; *n* = 5 rechallenge). Controls support OMV effects; no CpG-loaded uncoated control limits membrane-specific attribution.	[172]

**Table 6:** Representative cancer cell membrane-coated nanoparticles (CCM-NPs), including cargo/core, cancer applications, experimental models, administration regimens, comparators, reported outcomes, and evidence limitations.

Nanoplatform and Cargo/Core	Application	Experimental Model In Vitro and In Vivo	Route and Regimen	Comparators and Uncoated Core	Reported Outcomes	Evidence Level and Membrane Contribution	References
CSiFePNs: paclitaxel/Fe_3_O_4_-loaded mesoporous silica coated with MDA-MB-231 cell membrane (~220 nm).	Breast cancer; chemo and magnetic heating.	In vitro: MDA-MB-231 breast cancer cells. In vivo: not evaluated.	Cell incubation ± AMF: 0.60 mg Fe/mL, 24 h (captions: 48 h). Solution heating: 20–100 mg/mL, 100–300 W, ≤60 min.	Free PTX; uncoated drug-loaded SiFePNs; coated drug-free CSiFeNs; formulations ±AMF. MSN and Fe_3_O_4_ assessed for cell compatibility.	Enhanced uptake vs. uncoated core (3.76-fold fluorescence vs. free PTX at 4 h); strongest cytotoxicity with AMF; solution heating to 60 °C at 300 W/60 min.	In vitro only; replicates unspecified. Uncoated control supports membrane effects; IC50 reporting and exposure duration are inconsistent. No animal efficacy/safety data.	[198]
DOX/MSN@CaCO_3_@CM: DOX-loaded mesoporous silica with a pH-sensitive CaCO_3_ gatekeeper and LNCaP-AI cell membrane (~154 nm).	Prostate cancer; targeted, pH-triggered chemo.	In vitro: LNCaP-AI, MCF-7 and QSG-7701 cells. In vivo: subcutaneous LNCaP-AI tumors in BALB/c nude mice.	IV 100 µL; DOX 5 mg/kg on days 0, 4 and 8; 14-day follow-up.	PBS; free DOX; empty coated MSN@CaCO_3_@CM. No uncoated drug-loaded efficacy control. DOX/MSN@CM used to assess gatekeeper-dependent release.	Homotypic uptake and acid-triggered release (~70% at pH 5; 2.7% at pH 7.4/48 h). Increased apoptosis; tumor weight reduced 71% vs. PBS (free DOX: 38%); reduced Ki67.	Single preclinical study (*n* = 5/group efficacy; *n* = 4 cytotoxicity). No uncoated loaded-core control limits membrane-specific attribution; short follow-up in nude mice.	[199]
H-MnO_2_-GOx-Ce6@C: hollow MnO_2_ loaded with Ce6, surface-linked GOx and B16-F10 membrane; core ~75 nm.	Melanoma; combined PDT and starvation therapy.	In vitro: B16-F10 cells under normal/anaerobic conditions. In vivo: B16-F10 tumors in BALB/c mice (as reported); recurrence model.	IV + laser; 14-day efficacy and 20-day recurrence follow-up. In vitro: 655 nm, 5 min; in vivo dose/laser regimen unspecified.	PBS; free Ce6 +light; coated GOx-only or Ce6-only nanoreactors. Uncoated H-MnO_2_-GOx-Ce6 evaluated for accumulation, absent from efficacy groups.	Enhanced tumor accumulation, O_2_ and ROS generation; 12.9% cell viability with light, strongest tumor suppression and no recurrence over 20 days; no obvious weight or organ histology changes.	Single preclinical study; group sizes unspecified. Uncoated comparison supports targeting; absent uncoated efficacy control limits membrane attribution; short follow-up.	[200]
NP-R@M-M: R837-loaded PLGA coated with B16-OVA membrane and lipid-anchored mannose (~160 nm).	Cancer vaccine; melanoma prevention and treatment.	In vitro: BMDCs; macrophages. In vivo: B16-OVA tumors/challenge in C57BL/6 mice; 4T1 challenge for antigen specificity.	Intradermal vaccine: weekly ×3 prophylaxis (2 mg PLGA/20 µg R837 per dose); therapy on days 4, 11, 18. ±IV anti-PD-1 (20 µg × 6); follow-up: 45 days.	PBS; uncoated R837-loaded NP-R; coated formulations ±R837/mannose in prophylaxis. Established tumors: vaccine alone, anti-PD-1 alone and combination.	Enhanced APC uptake, DC maturation and lymph-node retention; strongest B16-OVA prevention, no 4T1 protection. Vaccine + anti-PD-1: 3/6 mice tumor-free at day 45; monotherapies failed by days 25–27.	Single preclinical study (*n* ≥ 5 prophylaxis; *n* = 6 therapy). Controls support membrane/mannose effects in prophylaxis; no uncoated therapy control. OVA model limits generalizability.	[201]
AECM@PC7A: STING-activating PC7A coated with IFN-γ-treated, antigen-enriched tumor cell membranes (10:3 membrane:polymer; ~120 nm).	Personalized cancer vaccine; prevention and therapy.	In vitro: BMDCs/splenic DCs; OT-I T cells. In vivo: B16-OVA/B16-F10/MC38 (C57BL/6), CT26 (BALB/c), MDA-MB-231 (PBMC-humanized NSG); metastasis, rechallenge and postsurgical models.	SC prophylaxis: 100 µg membrane +30 µg PC7A × 3, 5 days apart. IT therapy: × 3, 5 days apart (membrane/dose: B16-OVA/CT26, 300 µg; MC38, 100 µg; B16-F10, 500 µg). Humanized model: IT 300 µg × 2.	PBS; untreated CM@PC7A; membranes ±PC7A; lysate/peptide-PC7A vaccines; alternative adjuvants. Mechanistic controls: B2m-knockout membranes and CD8 depletion.	Enhanced DC/CD8 responses; reduced metastases and distal tumors. B16-OVA: 4/10 tumor-free >80 days; MC38: 92.3% growth inhibition, 60% remission. Survivors resisted matched rechallenge; humanized tumors suppressed.	Single preclinical study (*n* = 5–10/group efficacy). Controls support antigen enrichment/pMHC-I effects; no routine bare-PC7A therapy control. Humanized xenograft findings remain preclinical.	[195]

**Table 7:** Recent patents on membrane-coated nanoparticles for diverse therapeutic applications, highlighting publication details and key inventions.

Publication Date Month Year	Patent Jurisdiction	Membrane	Title	Summary of Invention	References
14.04.2020	China	Platelet–neutrophil hybrid membrane	Adriamycin-indocyanine green bionic nanoparticles and application thereof	Membrane-coated nanoparticles capture circulating tumor cells and tumor-derived exosomes, disrupting metastatic signaling. They enable synergistic chemo-photothermal therapy to eradicate primary tumors and inhibit breast cancer metastasis.	[204]
09.04.2024	China	Tumor cell membrane	Tumor cell membrane-coated mesoporous silica nanoparticles and their preparation method.	Membrane-coated mesoporous silica nanoparticles co-loaded with doxorubicin and 1-methyltryptophan enable targeted chemo-immunotherapy, offering high drug-loading capacity, good biocompatibility, and uniform dispersion.	[205]
17.12.2024	China	Tumor cell membrane	Membrane-coated PROTAC magnetic nanoparticles capable of enhancing technical efficiency of targeted protein degradation chimera as well as preparation method and application of membrane-coated PROTAC magnetic nanoparticles	Cell membrane-coated PROTAC magnetic nanoparticles enable targeted BRD4 degradation, MRI-guided tumor imaging, metabolic regulation, and enhanced antitumor efficacy against breast cancer.	[206]
28.11.2019	United States of America	Bacterial membrane	Modulating antibacterial immunity via bacterial membrane-coated nanoparticles	Bacterial membrane-coated nanoparticles provide targeted drug delivery and immunomodulation for the prevention and treatment of bacterial infections.	[207]
01.07.2022	China	Bacterial outer membrane vesicles	Preparation method of nano particles coated with bacterial outer membrane vesicles and having photo-thermal-chemotherapy-immune functions	Bacterial membrane-coated antimonene nanoparticles enable photoacoustic imaging and synergistic photothermal–photodynamic–chemo–immunotherapy for enhanced cancer treatment.	[208]

## Data Availability

No new data were created or analyzed in this study. Data sharing is not applicable to this article.

## Abbreviations

ABC	Accelerated body clearance
AUNCs	Gold nanoclusters
BCM-NPs	Bacterial cell membrane-coated nanoparticles
CCM-NPs	Cancer cell membrane-coated nanoparticles
CMCNPs	Cell membrane-coated nanoparticles
CSC	Cancer stem cell
DOX	Doxorubicin
EPR	Enhanced permeability and retention
EVs	Extracellular vesicles
iRGD	Internalizing RGD
MDR	Multidrug resistance
MGE	Metabolic glycoengineering
miRNAs	Micro RNAs
MPS	Mononuclear phagocyte system
MSCs	Mesenchymal stem cells
MSNs	Mesoporous silica nanoparticles
NIR	Near infrared
OMV	Outer membrane vesicle
PDT	Photodynamic therapy
PFT	Pore forming toxin
PM-NPs	Platelet membrane-coated nanoparticles
PTT	Photothermal therapy
PTX	Paclitaxel
RBCs	Red blood cells
RBC-NPs	Red blood cell-coated nanoparticles
ROS	Reactive oxygen species
RT	Radiotherapy
SCM-NPs	Stem cell membrane-coated nanoparticles
siRNA	Small interfering RNA
TNBC	Triple negative breast cancer
VDAs	Vascular disrupting agents
